# Extracting intersectional stereotypes from embeddings: Developing and validating the Flexible Intersectional Stereotype Extraction procedure

**DOI:** 10.1093/pnasnexus/pgae089

**Published:** 2024-03-19

**Authors:** Tessa E S Charlesworth, Kshitish Ghate, Aylin Caliskan, Mahzarin R Banaji

**Affiliations:** Kellogg School of Management, Northwestern University, Evanston, IL 60208, USA; Language Technologies Institute, Carnegie Mellon University, Pittsburgh, PA 15213, USA; Information School, University of Washington, Seattle, WA 98105, USA; Department of Psychology, Harvard University, Cambridge, MA 02138, USA

**Keywords:** gender, intersectionality, race, stereotyping, word embeddings

## Abstract

Social group–based identities intersect. The meaning of “woman” is modulated by adding social class as in “rich woman” or “poor woman.” How does such intersectionality operate at-scale in everyday language? Which intersections dominate (are most frequent)? What qualities (positivity, competence, warmth) are ascribed to each intersection? In this study, we make it possible to address such questions by developing a stepwise procedure, Flexible Intersectional Stereotype Extraction (FISE), applied to word embeddings (*GloVe*; *BERT*) trained on billions of words of English Internet text, revealing insights into intersectional stereotypes. First, applying FISE to occupation stereotypes across intersections of gender, race, and class showed alignment with ground-truth data on occupation demographics, providing initial validation. Second, applying FISE to trait adjectives showed strong androcentrism (*Men*) and ethnocentrism (*White*) in dominating everyday English language (e.g. *White + Men* are associated with 59% of traits; *Black + Women* with 5%). Associated traits also revealed intersectional differences: advantaged intersectional groups, especially intersections involving *Rich*, had more common, positive, warm, competent, and dominant trait associates. Together, the empirical insights from FISE illustrate its utility for transparently and efficiently quantifying intersectional stereotypes in existing large text corpora, with potential to expand intersectionality research across unprecedented time and place. This project further sets up the infrastructure necessary to pursue new research on the emergent properties of intersectional identities.

**Significance Statement**

Stereotypes at the intersections of social groups (e.g. *poor man*) may induce unique beliefs not visible in parent categories alone (e.g. poor *or* men). Despite increased public and research awareness of intersectionality, empirical evidence on intersectionality remains understudied. Using large corpora of naturalistic English text, the Flexible Intersectional Stereotype Extraction procedure is introduced, validated, and applied to Internet text to reveal stereotypes (in occupations and personality traits) at the intersection of gender, race, and social class. The results show the dominance (frequency) and halo effects (positivity) of powerful groups (*White*, *Men*, and *Rich*), amplified at group intersections. Such findings and methods illustrate the societal significance of how language embodies, propagates, and even intensifies stereotypes of intersectional social categories.

## Introduction

Since 2004, Google searches for the term “intersectionality” have increased exponentially, reaching a peak in February 2023 (SI Appendix). This increasing interest among the public parallels rising calls among social scientists to recognize how intersections of social group identities modulate group perception (1–3). Intersections of group identities, such as *race + gender*, produce unique and emergent stereotype content (4), as well as unique experiences of discrimination (5, 6) that would be missed if group identities were examined in isolation for experimental convenience. While social group intersectionality has received extensive humanistic analyses and qualitative theorizing (3, 7), there remains limited empirical evidence studying intersectionality at-scale (8). Today, methodological developments in testing and quantifying stereotypes in naturalistic language make such an effort possible (8).

In this study, we address past methodological limitations and advance empirical research on intersectional social group stereotyping by introducing a new stepwise procedure—the Flexible Intersectional Stereotype Extraction (FISE) procedure. The procedure is flexible in that it can be: (i) applied to large-scale naturalistic data of word embeddings trained from any text source, including different languages, geographies, or demographic groups; (ii) applied to any group intersection, so long as the group concept can be represented in words; and (iii) quantified in systematic and comparable metrics, such as the frequency (number) of traits associated with an intersectional group, to facilitate direct comparisons of intersectional stereotypes across diverse settings.

## Past research: extracting single group stereotypes from static embeddings

The FISE procedure builds from an emerging body of research using Natural Language Processing, and especially static word embeddings, such as *GloVe* (9), to propel the understanding of how social attitudes and stereotypes prevail in large-scale naturalistic data (10–12). For instance, static word embeddings have been used to quantify the relative relationships between single groups (e.g. *White*, *Black*) and attributes (e.g. *good*, *bad*), ultimately revealing that static word embeddings capture biases similar to attitudes obtained directly from human minds (13). Such findings from static word embeddings have been crucial in expanding understanding of topics ranging from how English language–based stereotypes have shifted across 200 years of history (12) to how social class is conceived in contemporary English (14) and to how gender stereotypes emerge in children's books (15).

Despite the insights gleaned from these studies, however, past work has largely represented each social group concept independently, thereby leaving stereotypes at *intersections* of identities unexamined. Such limitations occur, in part, because social science researchers often rely on the availability and ease of use in static single word embedding which have key advantages of being flexibly trainable across languages (16), time points (17), and diverse demographics (18). Still, static word embeddings arguably encountered difficulties for studying intersectional stereotyping because they cannot represent multiword sentences, and thus, it was difficult to conceive of how to represent intersections, such as *Black Women* and *White Men* accurately.

## Past research: extracting group stereotypes from contextualized embeddings

Acknowledging such seeming methodological difficulties, computer science research moved to studying social group biases using *contextualized* embedding models (e.g. *BERT* (19)) with multiword sentences (20–22). For instance, Guo and Caliskan (21) studied intersectional stereotypes across gender and race/ethnicity by representing group concepts in sentences using examples from everyday English language (e.g. “This is Aisha” or “Look at Keisha,” for example, sentences with Black woman names). As in previous static embedding analyses, the authors then computed relationships (cosine similarities) between the intersectional group–related sentences and positive/negative attribute sentences. Results showed evidence of intersectional emergence, in which new traits emerged as associated with the intersectional group that were not associated with the individual parent groups in isolation; such findings were further validated against known emergent content, providing confidence in the general approach of using language to study intersectional stereotyping.

Yet even contextualized language models can suffer from limitations when studying intersectional stereotypes. For instance, research with contextualized methods has to-date represented groups through first/last *names* (20, 21). Yet many social group dimensions are simply not encoded in names, especially more concealable groups, like sexual orientation, religion, or more physical stigmas such as body weight, age, or disability status (23). Additionally, given the greater computational and expertise demands of training or fine-tuning contextualized language models on new text corpora, social science research remains limited in its ability to examine the variability and scope of intersectional stereotypes if required to rely only on large language models (LLMs).

## The current research: FISE

To overcome such barriers for social science researchers, the current research introduces the FISE procedure as a flexible and transparent procedure that can be applied to both static and contextualized models, as necessary. We introduce FISE with a case studying focusing on intersections across three major social categories—race (in this case, Black/White, because of the dominance of Black/White relations in US English contexts), as well as social class (rich/poor), and gender (men/women)—that can shape group perception and its consequences of preference and discrimination (24, 25). In our primary analyses, FISE is applied across multiple pretrained embedding models of large-scale English text corpora, ranging from static *GloVe* embeddings trained on 840 billion words from the Common Crawl to contextualized *BERT* embeddings trained on a combination of Wikipedia and Common Crawl text. Additionally, in supplemental material and open code resources, we illustrate the flexibility of generalizing FISE to applications across non-English languages (French) for future researchers to address the English-centric focus of existing work (26). In supplemental material, we also generalize FISE to more complex three-way intersections of social groups (e.g. simultaneously crossing gender, race, and class) and show the advantages of FISE over simpler averaged vector approaches.

**Fig. 1.**
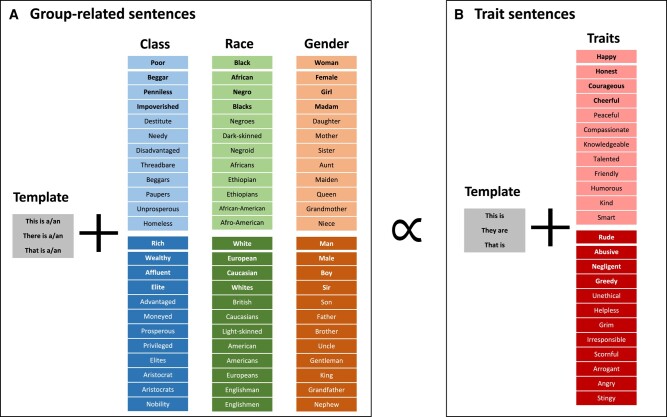
Group words used to represent class, race, and gender for applications of FISE. In the static embeddings, our primary analyses, FISE uses only the individual words for each group (no templates, in gray). In the contextualized setting (study 2 only), FISE is performed by looking at the cosine similarity (essentially a correlation) between target group-related sentences (A) and trait attribute sentences (B). A group sentence is created by first selecting a template (e.g. “This is a”), and then adding the group labels for class (e.g. “rich”), race (e.g. “Black”), and gender (e.g. “woman”). The process is repeated across all possible combinations, yielding 5,184 sentences from three templates and 12 words for each group (3 × 12 × 12 × 12 = 5,184). Similarly, for traits, all trait templates are combined with all positive/negative trait words, to yield a sample of 300–900 trait sentences, depending on how many traits are chosen. Nationality terms under the race categories (e.g. American) were chosen given the conflation of nationalities with racial groups in stereotypes and documented evidence that American = White.

To begin, for the simplest case of static embeddings and two-way intersections, FISE proceeds in five steps (see details in Methods and SI Appendix). First, we create a list of target concepts for occupation labels (study 1) or trait adjectives (study 2), as well as lists of group labels that represent each of the three groups of interest (gender, race, and class; Fig. 1). Second, we compute the cosine similarity of each target occupation/trait with each group concept (e.g. *janitor-White*, *janitor-Black*, and so on). Third, we take the difference of the cosine similarities along a group dimension (e.g. *janitor-White* vs. *janitor-Black*) and repeat this for the other group dimensions (e.g. *janitor-Rich* vs. *janitor-Poor*, *janitor-Man* vs. *janitor-Woman*). Fourth, we cross two group dimensions (e.g. race-by-class), placing each occupation/trait in the resulting *x-y* coordinate space according to its cosine similarity with the individual group dimensions. Fifth, we analyze the resulting number (frequency) and qualities (e.g. valence) of the occupation/traits in each quadrant.

As noted above, the goal of FISE is primarily to improve the *flexibility* for extracting intersectional content across any text source and any group intersection, with comparable, quantitative metrics. Crucially, given wide-ranging scholarly interest in intersectionality, the FISE method is also designed to be easy to use, transparent to understand, and low in computational demands so that it can be easily adopted by scholars from any field. Indeed, all codes and data are provided openly with clear guidelines to apply the method to the complexity of different group targets (sampling from the large space of potential group topics (27), including groups that may have concealable identities not encoded in names), as well as different languages, media sources, and even timespans.

## Study 1: intersectional stereotypes of occupations

The content and frequency of intersectional occupation stereotypes have received almost no consideration in large language corpora (28) even though it is known that workplace experiences of discrimination can be amplified for intersectional identities (29, 30). Moreover, occupational stereotypes provide an ideal case study because they can be compared with ground-truth data of actual occupation demographics to validate the FISE procedure. Thus, as a first introduction to FISE, we identify (i) how many and (ii) which occupations, from a list of 143 occupations from the 2022 *Bureau of Labor Statistics* report, are associated with each intersectional quadrant across race-by-gender, class-by-gender, and race-by-class.

## Results

### Frequency of intersectional occupation stereotypes in FISE vs. ground truth

How many occupations are associated with each intersectional quadrant in English language and in occupational demographic data? If FISE accurately identifies the frequency of intersectional occupation stereotypes (i.e. how many occupations are associated with each group), then FISE should show that intersectional groupings associated with the most occupations in the real world (e.g. *White Men*) are also associated with the most occupations in language. That is, if an intersectional group dominates the labor force in vivo, it should also dominate in the frequency with which it occurs in naturalistic language.

**Fig. 2.**
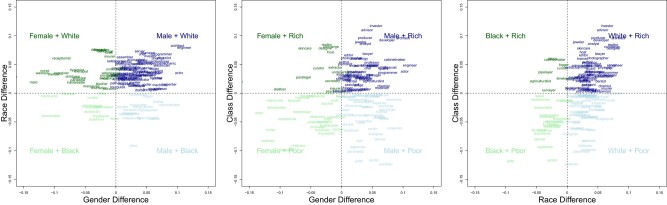
English language–based occupation stereotypes from FISE applied to static embeddings. Each panel represents the specific occupations associated with each intersectional quadrant in the contrast of (A) gender-by-race, (B) gender-by-class, or (C) race-by-class. Interactive scatterplots are available to zoom in on specific quadrants: https://osf.io/b9nmd/. Analogous plots for the other five methods and data sources are provided in SI Appendix.

Indeed, the dominance of an intersectional grouping in real-world data is mirrored in the number of occupations associated with each intersectional quadrant in English language (Fig. 2; Table 1). Chi-square tests confirm that actual frequencies of occupations are not significantly different from the associations extracted in language: for race-by-gender, χ^2^ = 7.53, *P* = 0.06, *V* = 0.19, for race-by-class, χ^2^ = 2.63, *P* = 0.45, *V* = 0.11, and for gender-by-class, χ^2^ = 5.46, *P* = 0.14, *V* = 0.17. For example, looking at actual occupation data we see that 48% of occupations are relatively more White (>50% point difference in White vs. Black representation) and occupied by Men (>50% men). In parallel, looking at language associations, *White Men* are linguistically associated with 59% of occupations, which, although descriptively higher than the actual representations, is not significantly different (see chi-squared tests above). Similarly, in real-world data, only 5% of occupations are relatively more Black and occupied by Women and, in language, *Black Women* are associated with 9% of occupations. Such accuracy is also found for the class-by-gender and race-by-class comparisons (Table 1). Ultimately, the language models show accuracy in identifying which intersectional groups dominate (are most frequent) in the occupational stereotype space and, conversely, which intersectional groups are made invisible (are least frequent) in occupational stereotypes.

**Table 1.** Percentage of occupations in each intersectional grouping (quadrant) for static embeddings and ground-truth data (non-*z*-scored).

Intersectional grouping	Ground-truth categorizations (BLS)	Static embeddings (GloVe CC 840B)
White + Rich	**53**	**46**
White + Poor	33	33
Black + Rich	2	6
Black + Poor	13	15
Male + Rich	**33**	**42**
Male + Poor	24	29
Female + Rich	21	10
Female + Poor	21	20
Male + White	**48**	**59**
Male + Black	10	12
Female + White	37	20
Female + Black	5	9

Occupation frequencies represent the *percentage* of occupations (out of 143 possible occupations) that are associated with each intersectional grouping (e.g. the *White Men* grouping is associated with 59% of occupations in language). Bolded numbers indicate the highest relative percentage for each intersectional quadrant.

### Which occupations are associated with intersectional groupings in FISE vs. ground truth?

Looking beyond *how many* occupations we next ask: *which* specific occupations fall in each quadrant (see Fig. 2)? To test accuracy here, we compare the “hits” (i.e. both language and ground truth classify an occupation as, for example, *White Rich*) to “misses” (i.e. language and ground truth deviated for specific occupation classifications). Across all 143 occupations, language and ground-truth categorizations showed a 57%, 47%, and 51% “correct hit” rate, for gender-by-race, gender-by-class, and race-by-class, respectively, which were not significantly different from chance, χ^2^ = 2.27, *P* = 0.13; χ^2^ = 0.28, *P* = 0.59; and χ^2^ = 0.03, *P* = 0.86. Crucially, however, two additional analyses show that, when sufficient signal exists in the real-world, FISE can in fact accurately capture occupational stereotypes.

First, we inspect the individual occupations that “missed” classification: as a few examples, *judge*, *analyst*, *accountant*, and *bartender* were classified as *White Men* occupations in English language data but, in ground-truth, were relatively more associated with *White Women.* Crucially, in ground truth, these occupations were around the 50% mark of men/women representation, with 55–59% women workforces, and thus provided ambiguous gender signals in ground-truth data. Similarly, occupations that were more ambiguously White/Black (e.g. with smaller differences in White/Black representation such as *guard*, *caregiver*, *recycler*) were more likely to be misclassified along race. Those that were more ambiguously Rich/Poor (e.g. around the median earnings; *investigator*, *librarian*, *plumber*) were more likely to be misclassified across class (i.e. as a “rich” occupation, above median earnings, or as a “poor” occupation, below median earnings).

Thus, in a second analysis, we looked at only those occupations that had an arguably clear real-world signal, using stricter criteria determined by the first and last author (specifically, <30% women in the BLS data was required for a “Men” classification; >70% women was required for a “Women” classification; see *SI Appendix*). Using only that subset of occupations with clear real-world signal on gender, race, and class, we found significant above-chance accuracy for all three contrasts of gender-by-race, 69% [53%, 82%], χ^2^ = 5.36, *P* = 0.02, gender-by-class, 70% [51%, 84%], χ^2^ = 4.36, *P* = 0.04, or race-by-class, 70% [50%, 86%], χ^2^ = 3.70, *P* = 0.05. In summary, when the real-world signal is clear, the language will accurately identify the occupations; when real-world signal itself is around chance then the language will accurately reflect such ambiguity.

## Study 2: intersectional stereotypes of trait adjectives

Study 1 shows that FISE can accurately identify both how many (the relative frequency) and which (the extreme classifications) occupations are stereotyped into intersectional social group quadrants across race, gender, and class. Thus, in Study 2 we are on firmer ground to expand the scope to consider intersectional stereotypes with trait adjectives, which provide no ground-truth data (i.e. we have no objective data on whether men or women, rich or poor, Black or White are more *honest* or *hardworking*). And yet, trait adjectives are the quintessential carriers of stereotypes and are consistently used in research on person or group perception. Study 2 also expands the research scope to demonstrate the flexible application of FISE across contextualized embedding models (*BERT* (19)), and identifies where conclusions converge or diverge across models, as well as what divergences teach us about how these models appear to represent language and society.

## Results

### Frequency of intersectional trait stereotypes

**Fig. 3.**
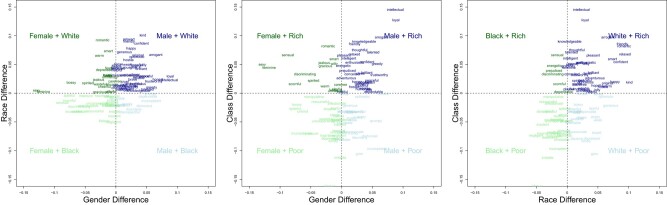
Language-based trait stereotypes from FISE applied to static embeddings. Each panel represents the specific traits associated with each intersectional quadrant in the contrast of (A) gender-by-race, (B) gender-by-class, or (C) race-by-class. Interactive online scatterplots are available to zoom in on specific quadrants and traits: https://osf.io/b9nmd/. Analogous plots for the other five methods and data sources are provided in SI Appendix.

In the primary case of static embeddings, there is clear evidence of both androcentrism and ethnocentrism, such that any intersection, including *Men* (vs. *Women*) or *White* (vs. *Black*), dominates in English language (Fig. 3; Table 2). Indeed, the highest relative frequency of traits occurred for *White Men*, associated with 59% of traits; the lowest relative frequency of traits occurred for *Black Women*, which was associated with only 5% of traits (Table 2). As reported in the SI Appendix, the imbalances in trait frequencies (e.g. the dominance of *White Men* over *Black Women*) deviated significantly from chance, with effect sizes ranging from *V* = (0.26–0.56), equivalent to small-to-moderate effect sizes. Thus, English language reveals evidence of intersectional dominance for powerful groups and intersectional invisibility for subordinate groups.

**Table 2.** Percentage of traits in each intersectional grouping (quadrant) across embedding methods (non-*z*-scored).

	Static embeddings			Contextualized embeddings		
Intersectional grouping	GloVe CC 840B	GloVe Wiki 6B	fastText Wiki 2M	Single words with template	Pooled words with template	Pooled words no template
White + Rich	**57**	**43**	**34**	**59**	**69**	**43**
White + Poor	32	24	30	28	22	29
Black + Rich	5	6	9	1	0	3
Black + Poor	6	24	27	12	9	25
Male + Rich	**44**	**33**	23	22	30	16
Male + Poor	21	23	**32**	11	7	20
Female + Rich	18	16	20	**38**	**39**	**30**
Female + Poor	17	28	25	29	24	34
Male + White	**59**	**46**	**40**	26	35	27
Male + Black	6	10	15	7	2	9
Female + White	30	21	24	**61**	**56**	**45**
Female + Black	5	23	21	6	7	19

Trait frequencies represent the percentage of traits (in this case out of 100 possible traits) that are associated with each intersectional grouping (e.g. the *White Men* grouping is associated with 62 out of 100 traits, whereas the *Black Men* grouping is associated with 3 out of 100 traits). Frequencies are compared within a data source (e.g. *GloVe CC 840B*) but across groupings, such that all four groupings within a data source will add up to 100. Bolded numbers indicate the highest relative percentage for each intersectional quadrant (e.g. 62% is bolded to reflect that *White Men* is the grouping with the highest relative percentage of traits across all groupings for that data source). The three contextualized embedding columns indicate three methods for extracting embedding vectors for groups. As described in the main text, *single words with templates* indicates that a vector is created from averaging across the hidden state vectors for the template (e.g. “This is a”) and the first subtokens of group words (e.g. “rich African woman”); *pooled words with templates* indicates that the vector is created from averaging across the hidden state vectors for the template and the pooled group words (pooled across subtokens; e.g. “rich African + American woman); pooled words no template indicates that the vector is created from the first four layers of the hidden state vectors pooled across only the group words (e.g. rich African + American woman).

Interestingly, the data show less support for class-centrism, i.e. rich does not dominate frequencies in language to the same extent that White supersede Black, or men supersede women. For example, *Black Poor* (6% of traits) and *Black Rich* (5% of traits) are similar in frequency showing that the low frequency of traits associated with *Black* is not altered even after including the dominant class group *Rich*. Perhaps class may be less of a marked category in language: we may be unlikely to point out that someone is *rich*, unless it is extreme wealth, because categorizing class is prone to subjective judgments of wealth cues (31). In contrast, race and gender may be relatively less ambiguous in categorizations and therefore more likely to be noted in language and to shape trait frequencies.

### Qualities of intersectional trait stereotypes

As in study 1, our next analyses go beyond the overall *number* of traits to consider the specific traits and their qualities (Table 3). Specifically, we consider six qualities of traits that are of foundational interest to group perception (25): (i) valence; (ii) dominance; (iii) arousal; (iv) warmth; (v) competence; and (vi) commonality (how often a given trait is used in everyday contemporary English). To examine such qualities, we ensure that each quadrant has a relatively similar representation in number of traits, as this control allows us to compute the relative qualities of intersectional groups independent of their imbalance of representation. Additionally, this control is required to ensure that even low-frequency quadrants (e.g. *Black Rich*, associated with only five traits) have sufficient representation to examine the relative qualities. Thus, we first mathematically adjust the frequencies across quadrants by *z*-scoring the placement of each trait along individual group dimensions and compute each quadrant's average scores of the traits” qualities. Crucially, even after re-scaling the number of traits across quadrants, the *features* of those traits continue to show intersectional differences (Table 3 for *GloVe 840B*; results for all embeddings in SI Appendix).

**Table 3.** Relative percentages of trait qualities (by valence, warmth, competence, arousal, dominance, commonality) within each intersectional quadrant for GloVe CC 840B static embeddings.

	Valence		Warmth		Competence		Arousal		Dominance		Word common.
Intersectional grouping	Positive	Negative	Warm	Cold	Comp	Incomp	High	Low	High	Low	
White + Rich	78	22	81	19	78	22	48	52	78	22	4.13
White + Poor	26	74	26	74	26	74	48	52	26	74	3.88
Black + Rich	69	31	69	31	69	31	46	54	65	35	3.56
Black + Poor	21	79	21	79	25	75	58	42	21	79	3.41
Male + Rich	70	30	70	30	70	30	47	53	70	30	4.01
Male + Poor	28	72	28	72	28	72	56	44	28	72	3.78
Female + Rich	78	22	83	17	78	22	48	52	74	26	3.63
Female + Poor	18	82	18	82	23	77	50	50	18	82	3.48
Male + White	56	44	56	44	56	44	53	47	56	44	4.15
Male + Black	43	57	43	57	43	57	48	52	43	57	3.57
Female + White	50	50	56	44	50	50	39	61	50	50	3.77
Female + Black	48	52	48	52	52	48	56	44	44	56	3.42

For all columns except the last one, numbers reflect the percentage of traits from the given quadrant (e.g. from the intersectional grouping of *White Rich*) that are, for example, coded as positive (vs. negative). The “word common” column reflects the average Zipf score across all traits in that quadrant. Zipf scores are the base-10 logarithm of the number of times a word appears in a billion words. For instance, Zipf = 4.13 for the *White Rich* quadrant reflects that the traits associated with this quadrant have occurred in language 10^4.14^ times, or ∼13,490 times, for every 1 billion words.

**Fig. 4.**
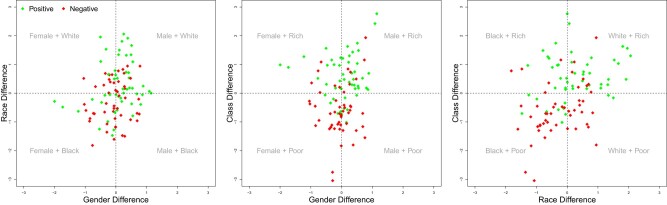
Valence of traits (after *z*-scoring) associated with intersectional comparisons of (A) gender-by-race, (B) gender-by-class , and (C) race-by-class. Traits are color-coded by valence. All figures use data from the *GloVe Common Crawl 840-billion* data source of static embeddings. Analogous plots for the other five methods and data sources are provided in supplemental material.

First, intersections differed in their relative proportion of positive/negative traits (Fig. 4 and Table 3). For example, 78% of traits associated with *White Rich* were positive, while only 21% of traits associated with *Black Poor* were positive. In general, valence imbalances appeared to be the largest across class (Rich/Poor).

Second, traits differ in word arousal (degree of “activity” evoked by the trait) and dominance (degree of “control” evoked by the trait (32)) as well as qualities of warmth/coldness and competence/incompetence (25). For warmth/coldness, competence/incompetence, and dominant/nondominant traits, results paralleled valence: intersections including powerful groups of *White*, *Rich*, or *Men* were not only more likely to be positive, but were also more likely to be associated with traits expressing warmth, competence, and dominance (Table 3). As one example, for *Rich White*, 81% of traits were warm, 78% were dominant, and 78% were competent. In contrast for *Poor Black*, 79% of traits were cold, 79% were subordinate, and 75% were incompetent. Such findings emphasize the “halo effects” (i.e. the unwarranted generalization of positivity across multiple dimensions (33)) that become amplified for powerful intersectional groups. Only the dimension of arousal did not differentiate across intersectional groups, perhaps because this dimension is often seen as a less central dimension of meaning (32), and thus may not be as relevant to distinguish between social categories.

Finally, intersectional social group quadrants also differed in how common the associated traits were in everyday language, using commonality scores (as Zipf scores, see Methods) taken from the *wordfreq* norms in Python (34). Traits associated with any *White*, *Men*, or *Rich* intersection were more commonly used in language than traits associated with any *Black*, *Women*, or *Poor* intersection (Table 3). For instance, the traits associated with *Rich Men* had, on average, 10,232 occurrences per billion words (Zipf = 4.01, equivalent to 10^4.01^). In contrast, the traits associated with *Poor Women* had, on average, 3,020 occurrences per billion words (Zipf = 3.48, or 10^3.48^ occurrences), corresponding to roughly one-third the commonality of *Rich Men* traits. Imbalances in commonality reveal further andro-, ethno-, and class-centrism in language. When it comes to our most common traits (e.g. *friendly*, *happy*, and *smart*), powerful groups dominate; subordinate groups, in contrast, are relegated to description with more idiosyncratic traits (e.g. *manipulative*, *spiteful*, and *spirited*) that may, in turn, contribute to perceptions of nonprototypicality.

### Differences between static and contextualized embeddings

So far, we have focused on describing the general pattern of results for static embedding models, which remain the most widely used method in the social sciences. However, given the flexibility of FISE procedure, we can also compare results across contextualized embeddings from BERT (Table 2). That is, we can compare the results of the relative frequencies of traits classified into intersectional quadrants (e.g. how many traits are classified as associated with *White Men*) not only across multiple static embeddings from various algorithms (*fastText* and *GloVe*) and data sources (*Wikipedia*, *Common Crawl*) but also across contextualized embeddings with additional variations in embedding extraction methods (SI Appendix).

The frequencies of classified traits across six different models and methods are listed in Table 2. Chi-squared tests evaluating how similar these frequencies are across methods showed small overall effect sizes, implying that static and contextualized models generally reveal similar patterns of frequencies: for race-by-class, *χ*^2^(15) = 59.02, *P* < 0.001, *V* = 0.18; for gender-by-class, *χ*^2^(15) = 64.23, *P* < 0.001, *V* = 0.19; and for race-by-gender, *χ*^2^(15) = 100.03, *P* < 0.001, *V* = 0.24.

Nevertheless, we identify one seemingly systematic difference between results for static and contextualized embeddings when it comes to the gender-by-class and gender-by-race comparisons. For static embeddings, whenever gender was included, the greatest frequency (as well as positivity, commonality, etc.) was associated with the default group (e.g. *Rich Men*) (35). Such patterns of “default” group dominance reflect the ground truth of how groups dominate in the world (see study 1). For contextualized embeddings, however, the greatest frequency (and positivity and commonality) was associated with the intersectional grouping that joined female (a low-power group compared with male) with a higher power class group (e.g. *Rich Women*). Perhaps, in contextualized embeddings, group information is considered more jointly since the group labels are provided together in a single sentence. Thus, seemingly incongruent pairings of high-power female groups are more easily noted and “marked” in the model (8). This marking could increase the frequency of traits associated with the group.

## General discussion

Computational and social sciences have both increasingly recognized the need for intersectional approaches to understand social biases and their impacts (7, 21, 36). And yet, despite the call for more intersectional research there remains, to our knowledge, no simple, flexible, and transparent approach to quantifying intersectional stereotype content, at-scale in massive language data. In this study, we introduce such a method for interdisciplinary research—the FISE procedure. We apply FISE to a first case study of intersectional stereotypes across occupations and personality traits at the intersection of gender, race, and social class. A primary aim was to illustrate the kinds of empirical insights that can come from this new methodological approach. Although many of the nuanced findings about individual groupings, traits, and occupations remain beyond the scope of this first analysis, we emphasize four overarching empirical conclusions that can help propel intersectional analyses: (i) the alignment of intersectional stereotypes in English language with ground-truth US data, emphasizing how language both reflects and reinforces social patterns; (ii) the language dominance (frequency) of powerful intersectional groups, as well as the relative invisibility of subordinate intersectional groups; (iii) the “halo effects” that surround powerful intersectional groups, especially those involving high social class; and (iv) informative divergences (and convergences) between static and contextualized language models.

### Frequencies of intersectional occupational stereotypes: alignment with ground-truth data

In study 1, we sought to validate FISE by showing that results of intersectional occupation stereotypes identified from English language align with ground truth on actual occupational demographics of those occupations in the United States. In addition to the relevance of occupational stereotypes for understanding workforce discrimination in hiring and pay (29, 37), occupational stereotypes also provide a necessary setting for validating a new method because they have associated ground-truth data on demographic representations. Indeed, classifications of 143 occupations into their intersectional quadrants showed evidence that FISE validly identifies true signal from real-world data. First, intersectional quadrants that dominated in the real-world data (e.g. *White Men* were associated with the most occupations in the real-world) also dominated in language associations. Second, when specific occupations provided arguably clear signal in ground-truth data (e.g. they were >70% female and >70% White), language associations correctly classified the occupation into the specific intersectional quadrant (e.g. as *White Women*). When specific occupations provided more ambiguous signal (e.g. female representation was ∼50%), the language also showed that ambiguity (e.g. showed incorrect classifications of gender), thus providing an accurate reflection of the signal in reality.

Beyond validation of FISE, such results also illustrate new insights and opportunities for future research on the intersectionality of workforce demographics. For instance, as elaborated below, the dominance of a group in language can, in turn, feed back into legitimizing the group's dominance in the world (38). The dominance of *White Men* or *Rich Men* in the language of occupations may hamper other strategies to create change in workforce demography (39). Given the unique flexibility of FISE for application across historical language (compared with previous approaches that were less generalizable), future research can, for the first time, examine questions including how occupation stereotypes in language hinder or help changes in workforce demographics.

### Frequencies of intersectional trait stereotypes: the dominance of powerful groups

Having provided initial validation of the accuracy of FISE, we turned to trait-based stereotypes, a domain that has no ground-truth data but underlies the content of most group stereotypes (25, 40). These frequencies of traits uncovered clear patterns of androcentrism, or the dominance of *Men* in everyday culture and language, a finding that we and others have documented and discussed, albeit at smaller scales (41, 42). However, the current work is among the first to also emphasize the extent of ethnocentrism, or the dominance of Whiteness in Internet text. Such findings add new quantitative evidence to past qualitative theorizing on the White “default” in culture (43) and show that it is of the same scale as the masculine defaults seen in previous work.

The dominance of both Men and Whiteness in language patterns has downstream consequences in Artificial Intelligence (AI) and language applications (e.g. in machine translation, text classification, and text generation). Indeed, so much of the observed bias in AI more generally seems to be a result of imbalances in the training data. For example, AI-generated faces are judged as more realistic than natural human faces, but only for *White* faces due to the dominance of White faces in training data (44, 45). Moreover, androcentric and ethnocentric dominance can also serve to reify mental stereotypes about which groups *should* dominate in society. For example, experimental research has shown that the frequency of women vs. men in Google search outputs shapes beliefs about the default “person” as well; cultures that have greater male-dominated search outputs also more strongly endorse androcentric defaults (38). In short, the current results may play into a feedback loop in which language both reflects and reinforces existing social imbalances—imbalances that are magnified in intersectional comparisons (6, 46).

### Qualities of intersectional trait stereotypes: halo effects

Beyond frequency, we also consider the intersectional differences in the *qualities* of the stereotype content, specifically the average positivity, warmth, competence, dominance, and commonality (i.e. how common the traits are in everyday language use). The results show that any intersection that included *Rich*, and, to a lesser extent, *White* or *Men*, consistently anchored the positive end of the spectrum (e.g. positive, warm, and competent). Notably, class was the strongest dimension determining the qualities of traits, with *Rich* generating “halo effects” (47) across all qualities. This may be surprising considering explicit measures suggesting that stereotypes of Richness often generate mixed content (e.g. competent but also cold (25, 48)). However, evidence of “halo effects” are especially likely to be observed with relatively indirect measures of stereotyping (33, 49), as in the current research.

### Comparison of static and contextualized models

As its name suggests, the primary advantage of the FISE procedure is its flexibility for application across any embedding model, including both static (*GloVe*) and contextualized models (*BERT*) trained on diverse Internet data sources. Critically, emphasizing the robustness of primary conclusions, results within all static models were consistent with one another (i.e. all showed similar patterns of the relative frequencies and features of traits), and results within all contextualized embedding approaches were consistent with one another. Additionally, static and contextualized models agreed for frequencies and features of traits in the class-by-race contrast, with *White Rich* consistently associated with the most traits and the most positive traits, regardless of method.

At the same time, however, the few divergences between static and contextualized approaches were also informative. Static embeddings seem to capture the dominance of “default” powerful groups in language (e.g. *White Men*) in which both group dimensions (e.g. gender and race) reflect high power. Contextualized embeddings, however, seem to capture the “markedness” (50) of incongruent nondefault groups (e.g. *White Women* or *Rich Women*), where one group dimension contains power (e.g. *White* and *Rich*), but the other group (*Female*) is subordinate. Perhaps, the focus on “markedness” arises for contextualized embeddings because of the *joint* consideration of all group dimensions that might more readily highlight the unexpected (incongruent) group of “powerful females” (51). These differences between static and contextualized models can help researchers make informed decisions about which models to use based on their interests in default vs. markedness of subordinate groups. Additionally, future work can identify what methodological differences between static and contextualized embeddings help explain why default vs. marked gender groups are differentially emphasized across methods.

### Limitations

In supplemental analyses, we test and demonstrate the robustness of inferences across modeling approaches (static, contextualized) and additional specifications (how groups were represented, the numbers of traits, types of traits, verb, or noun lists; SI Appendix). Despite general patterns of consistency and robustness across such tests, the current inferences face other limitations. First, there are theoretical limitations: social group intersectionality is sometimes (but not always) thought of as an inherently contextualized, emergent phenomenon that is nonadditive (7, 36, 52). Therefore, the FISE approach of crossing two (independently created) axes from single dimensions of groups may not be an appropriate methodological assumption. While this is an inherent limitation for addressing some theoretical perspectives, we emphasize that the general results were broadly convergent across contextualized and static approaches. Thus, the FISE procedure (even applied to static embeddings) appears to be sufficient to approximate a contextualized process of intersectionality.

Additionally, FISE relies on representing group and attribute concepts in text. Especially in the case of static embeddings (where group concepts are lists of single words rather than contextualized sentences), the results remain limited by concerns around polysemy (e.g. *Black* and *White* also refer to colors), and frequency (e.g. some group words like *impoverished* are more rare than everyday words like *men*). Here and in previous work (12), we have attempted to rule out concerns of polysemy by using *lists* of words that, together, triangulate toward the intended group concept, and have shown that this list-based approach indeed captures the group concept rather than alternative polysemous meanings. Moreover, here we show that even two different ways of representing the group concepts (with lists of 4 or 12 words) yield similar overarching conclusions (SI Appendix).

Finally, we note that readers may question the relevance of FISE at a moment when the current static embeddings and BERT contextualized embeddings are surpassed by more sophisticated language model competitors. We see the approach as holding continued relevance because static embedding models will continue to be used for their wide availability, transparency, and flexibility for training on diverse (and relatively small) datasets. As such, demonstrating methods to use static and simple contextualized models for intersectional analyses is crucial. Although LLMs like GPT (Generative Pre-trained Transformer) are widely discussed and increasingly used, they suffer from serious limitations of nonrepresentative training data, as well as limited transparency (53). Nevertheless, we look forward to future work generalizing FISE to more sophisticated language models, including the newly developed contextualized construct representations argued to be more aligned with psychological representations (54).

More generally, we look forward to future research using FISE as interdisciplinary infrastructure for addressing long-standing questions of intersectional stereotyping, especially on the prevalence and features of emergent content (not contained in parent group stereotypes). With such tools, the study of intersectionality and emergence can be expanded in real-world language at an unprecedented scale, even across place, languages, demographics, and history.

## Methods

### Application of FISE to static word embeddings

Our primary test case is the set of pretrained *GloVe* embeddings trained on 840-billion-word tokens from the Common Crawl; replications for static embeddings are performed across *GloVe* embeddings trained on 6 billion tokens from Wikipedia, and *fastText* embeddings trained on Wikipedia. After choosing the text corpus, FISE proceeds in five steps. First, we identify a list of target concepts. In study 1, we use ∼150 occupation labels generated from the 2022 Bureau of Labor Statistics report (55); occupation labels, methods for generating them, and approaches to classify occupations into intersectional quadrants are detailed in the SI Appendix. In study 2, we use 100 traits drawn from a list of ∼400 available traits (56) but only the 50 most positive and 50 most negative traits based on ratings from Warriner et al. (57); replications with longer lists of 200 and 300 traits are reported in SI Appendix.

Second, for each occupation/trait concept (e.g. *kind*) we compute cosine similarities between the embeddings for the target word (*t_i_*) and all words representing a given group concept (*W_gA_*, e.g. *Rich*, which is represented by group words including *rich*, *wealthy*, *affluent*, and so on; see Fig. 1 for all words), and divide by the number of words in that group representation (*N_gA_*) yielding an average word-group A association (e.g. *kind-Rich* association; Eq. 1). We then repeat the procedure for the contrast group (e.g. *kind-Poor*). For analyses in which we use *z*-scored results, we standardize scores at this stage, taking each *t_i_* score, subtracting the mean across scores, and dividing by the standard deviation across all scores.


(1) $$t_{i(A)}=\;\frac{\sum \cos(W_{gA},\;t_{i})}{N_{gA}}.$$

Third, we take the difference between averaged cosine similarities to get the placement of the target word along the given group dimension (e.g. *kind-Rich* − *kind-Poor*  *=*  *kind-CLASS;* Eq. 2) and repeat for all other dimensions of groups (in this case, also gender and race, to also extract the *kind-GENDER* and *kind-RACE* placement).


(2) $$t_{i(AvB)}=t_{i(A)}-t_{i(B)}.$$

Fourth, we bring in the intersectional analysis by crossing two group dimensions (e.g. class and gender) in an *x-y* coordinate space and place each target word in that space according to its association with the respective group dimensions (e.g. *t_i_*_(*AvB*)_ represents the *x*-axis of *CLASS*, while *t_i_*_(*CvD*)_ represents the *y*-axis of *RACE*). This *x-y* space reveals the intersectional stereotypes associated with each of the four group intersections (e.g. *Rich Men*, *Rich Women*, *Poor Men*, and *Poor Women*). Fifth and finally, the frequency and qualities of these associated traits/occupations is analyzed.

### Application to contextualized word embeddings

The above procedure can be adapted to most cases in which embeddings are extracted for group and trait representations, including for contextualized embeddings, such as BERT. We demonstrate such an extension by adapting FISE for use with the *BERT base-uncased model* and three previously validated methods for retrieving embeddings of group representations from the model (described below).

**Fig. 5.**
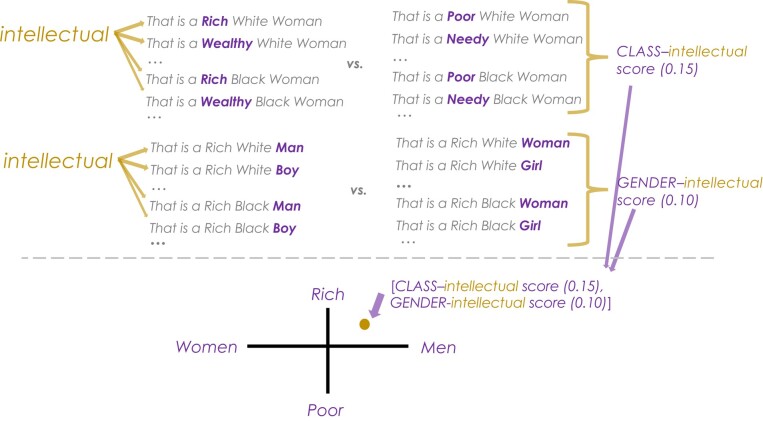
Illustration of the application of FISE. In the case of static embeddings, only the lower half of the figure applies: each target trait (e.g. *intellectual*) is associated with two single group dimension (e.g. *CLASS*, based on relative associations to *Rich* vs. *Poor*) and then placed in the intersectional *x-y* space according to its associations with the two group dimensions. In the case of contextualized embeddings, we use sentence prompts (top half of figure) and average across all instances of a given group concept (e.g. *Rich*) vs. the contrasting group (e.g. *Poor*) to arrive at the group dimension-trait association. As with the static embeddings application, the final step is to place each trait in the *x-y* space defined by the two group dimensions.

The FISE procedure for contextualized embeddings again follows five steps (Fig. 5). First, and different from static applications, group targets are operationalized using group *sentence* probes (Fig. 1) to include all three intersectional group identities at once (e.g. “This is a rich black man”) varying class (words representing rich/poor), race (words representing Black/White), and gender (words representing man/woman). These three group targets are placed in a semantically bleached template (e.g. “This is,” “That is,” and so on); templates are semantically bleached to ensure that the extracted embedding captures the signal associated with the main (in this case, group-related) stimulus. Attributes (in this case, we only use traits) are also operationalized using sentence probes with the traits placed in a semantically bleached template (e.g. “They are kind/unkind”).

Second, to extract associations of a target word with a single group dimension (e.g. class), we need to average across the other dimensions (e.g. gender, race) that were used in the original contextualized sentences. To that end, for each trait target word (e.g. *kind*), we compute the average cosine similarity to a given three-way target intersection (e.g. rich white men) as well as to the comparison target intersection that varies only on the group dimension of interest (e.g. *poor* white men, when we are looking at *CLASS*). We then take the difference between these two cosine similarities (e.g. *kind-rich white men − kind-poor white men = kind-CLASS white men* effect). We perform this for all contrasts along the dimension of interest to yield *kind-CLASS* effects for *White Men*, *Black Men*, *White Women*, and *Black Women*. We then average across these four contrasts for a single *kind-CLASS* association. Third, we repeat the above computations for a second group dimension (i.e. gender or race). Fourth, we place each target trait in an *x-y* coordinate space. And fifth, we perform all analyses of frequency and content.

### Extracting embeddings from contextualized models

While static embeddings provide the embeddings off-the-shelf, contextualized embeddings require an additional step to extract embeddings of a given sentence. For robustness, we employ three common approaches for extracting embeddings from the BERT model (58): (i) embedding templates (20); (ii) pooled embedding templates; and (iii) without templates.

First, “embedding templates” (20) extract embeddings of a given set of group words in our embedding template (e.g. “rich,” “African American,” and “woman” in the template “this is a”), by pulling the BERT embeddings of the first subtoken in the group words (e.g. “rich,” “African,” “woman”). Next, we use the hidden state vectors (only the top-most BERT layer) of those subtokens and average across the three group subtokens (rich, African, woman). This yields a single vector embedding for the BERT representation of the group target “rich, African American woman” as it is contextualized in the sentence “This is a rich African American woman.”

Second, “pooled embedding templates,” begins as above but, instead of taking the hidden state vectors of only the first subtoken for each group-related word, we instead take the mean pooled embeddings of *all* subtokens of the group-related words. To continue with our example, in the sentence, “This is a rich African American woman,” we first take the average across the hidden state vectors for “African” and “American” (the only target word with two subtokens) to create a single pooled embedding vector for “African American.” We then calculate the average across the pooled embedding of “African American” with the embeddings of “rich” and “woman” (which were only one subtoken and so did not need to be pooled) to again obtain a single vector representation of the group words in the contextualized sentence.

Third and finally, “without templates,” we move beyond templates, which, although argued to be semantically bleached, may nevertheless convey information that distracts from the target group representations. Instead of templates, we average over the pooled embeddings for just the three group words using the first four layers of the BERT model for each target word (59, 60). Consider again the example of assessing stereotypes to “rich African American woman,” in this setting we calculate the average of the pooled embeddings for three group words (“rich,” “African American,” and “woman”) across the first four layers of BERT. We do not include the embeddings of any template words in the final average.

### Robustness and sensitivity analyses

To verify the robustness and generalizability of our results for the more novel analyses of trait stereotypes, we test 6 variations of trait and group lists. In addition to (i) “Model 1: Full model,” in which we use the full 24 group words for each dimension (see Fig. 1) and the list of 100 traits, we also compute the following variations: (ii) “Model 2: Reduced group words” in which we change the group list to include only the 4 most central group-related words (bolded in Fig. 1); (iii) “Model 3: 200 traits” in which we extend the list of traits to include the top 100 most positive and top 100 most negative traits from the Peabody list; (iv) “Model 4: 300 traits,” as above but now with 300 traits total; (v) “Model 5: Nouns” in which we use parts-of-speech tagging and extract the top-100 positive/negative nouns (replacing the trait list); and (vi) “Model 6: Verbs” in which we change the trait list to top-100 positive/negative verbs.

As elaborated in SI Appendix, the primary empirical conclusions from study 2 were retained across model variations. First, regardless of (i) how many traits we used (100, 200, and 300), (ii) whether we represented groups with full or with reduced lists (of only 4 most central terms), and (iii) whether we used nouns, verbs, or the original trait lists, raw frequencies continued to show patterns of andro-, ethno-, and, to a lesser extent, class-centrism. Second, even after mathematically aligning frequencies using *z*-scoring, we found that the features of traits continued to differ across intersectional groupings, with relatively greater positivity, warmth, competence, and dominance for intersectional quadrants including *White*, *Rich*, or *Men.*

## Supplementary Material

pgae089_Supplementary_Data

## Data Availability

All data and analyses reported in this work are publicly available through https://osf.io/b9nmd/.
