# Supplementary material for: Extracting intersectional stereotypes from embeddings: Developing and validating the Flexible Intersectional Stereotype Extraction procedure
Source: PNAS Nexus. 2024 Mar 19;3(3):pgae089. doi: 10.1093/pnasnexus/pgae089 (PMC10949907; doi:10.1093/pnasnexus/pgae089)
Supplement: pgae089_Supplementary_Data [file pgae089_supplementary_data.docx]

***Supplementary Information for:***

*Extracting Intersectional Stereotypes from Static and Contextualized Embeddings*

Tessa E.S. Charlesworth^1^, Kshitish Ghate^2^, Aylin Caliskan^3^, Mahzarin R. Banaji^4^

^1^ Kellogg School of Management, Northwestern University

^2^ Carnegie Mellon University

^3^ Information School, University of Washington

^4^ Harvard University

All analysis scripts can be accessed at the project’s Open Science Framework page (view-only link for peer review): <https://osf.io/b9nmd/?view_only=f0b512840ad6488fb276cc3a48e09ddd>.

Table of Contents

[Introduction: Public interest in intersectionality 3](#_Toc154932673)

[Study 1: Creating occupation labels and ground-truth classifications 4](#_Toc154932674)

[Study 1: Ground-truth intersectional classifications of occupations 6](#_Toc154932675)

[Study 1: Comparisons between occupational language stereotypes and ground-truth data 8](#_Toc154932676)

[Study 1: Comparisons between occupational language stereotypes and ground-truth data for individual group dimensions 13](#_Toc154932677)

[Study 2: Comparisons to simpler (averaged vector) approach 15](#_Toc154932678)

[Study 2: Illustration of three-way race-by-gender-by-class intersectional analyses 18](#_Toc154932679)

[Study 2: Illustration of intersectional analyses for French embeddings 20](#_Toc154932680)

[Study 2: Relative features of traits for all embedding methods 22](#_Toc154932681)

[Study 2: Robustness across variations in number of traits 25](#_Toc154932682)

[Study 2: Robustness across variations in group lists 27](#_Toc154932683)

[Study 2: Robustness across nouns and verbs 31](#_Toc154932684)

# **Introduction: Public interest in intersectionality**

To illustrate the rising public interest in intersectionality we retrieved the relative frequencies of searching for the term “intersectionality” from Google Search Trends between 2004-2023 (data retrieved on September 26^th^, 2023). The y-axis of Figure S1 indicates the Google-normed relative interest, which has a maximum value of 100, indicating the month that had the highest overall interest. All other values are proportional to that maximum search rate (e.g., a score of 50 is approximately half of the overall maximum interest). Results show a curvilinear rise in interest over time, with the peak in February of 2023, and with the greatest interest since the second wave of the Black Lives Matter movement in summer 2020.

**Fig S1. Public interest in the term “intersectionality” across time (2004-2023); data from Google Search Trends.**

# **Study 1: Creating occupation labels and ground-truth classifications**

Data were retrieved from the Bureau of Labor Statistics (BLS) report 2022 data using Table 11 (<https://www.bls.gov/cps/cpsaat11.pdf>) and Table 39 (<https://www.bls.gov/cps/cpsaat39.pdf>). Table 11 provides detailed occupation statistics by sex, race, and ethnicity (used to calculate gender and race proportions); Table 39 provides detailed occupation statistics by median weekly earnings (used to calculate relative social class proportions).

First, to make the occupations usable with static single word embeddings, we reclassified as many occupation labels as possible into single words. For example, the original BLS label “Management, business, and financial operations occupations” became “manager”; “Financial and investment analysts” became “investor”; and so on. All conversions are provided in the “BLS_rawdata.csv” file on OSF and all final, cleaned data from the 150 occupations with available single labels is provided in the “BLS_cleaneddata.csv”. Of course, these conversions to single labels are imperfect and cannot fully capture the subtlety of many occupation descriptions; as such, we stress that readers take results with the caveat that low correlations or unexpected results for any given single occupation may arise, in part, from the ambiguity of translating occupations into single labels.

Second, to make relative group classifications (e.g., which occupations were male-dominated, female-dominated, and so on), we used the following criteria:

- For gender (men vs. women), we classified all occupations that had < 50% women as “men” occupations, and all those with $\geq$ 50% as “women” occupations.
- For race (White vs. Black), we first took the difference between the percent of the occupation workforce that was White and the percent that was Black, then we classified all occupations that were > 50 percentage points higher for White than Black as relatively “White” occupations, and all those with $\leq$ 50 percentage points White-Black as relatively “Black” occupations.
- For social class (rich vs. poor), we classified all occupations that had median weekly earnings > $1000 as “rich” occupations, and all those with median weekly earnings of $\leq$ $1000 as “poor” occupations.

As elaborated below, we also performed supplemental analyses with “stricter” criteria classifying by gender, race, and class to remove those occupations that sat around the 50/50 point (or a point of ambiguity in classification).

# **Study 1: Ground-truth intersectional classifications of occupations**

Figure S2 illustrates the individual occupations that are classified into each intersectional quadrant based on the criteria described above. Interactive scatterplots to zoom in on specific quadrants can be found on the project’s OSF page. The strongest occupations within each quadrant are those furthest from the origin. For example, in Panel A, the most *Male+White* occupations include *cabinetmaker*, *pipelayer*, and *pilot*; the most *Female+White* occupations include *hygienist, pathologist, fundraiser*; the most *Male+Black* occupations include *taxidriver, guard, barber;* the most *Female+Black* occupations include *caregiver, phlebotomist, manicurist.* Note that these latter occupations are those that are relative less-White, even if they may not be high percentage Black (instead they may be high percentage Asian, for example); nevertheless the fact that they are less-White results in a smaller White-vs.-Black gap in representation and thus places them in the relative “Black” or non-White quadrant.

| **A.** | ** |
| --- | --- |
| **B.** | ** |
| **C.** | ** |
|  |  |

**Fig S2. Scatterplots of real-world demographic representation classifications of 143 occupations.** Panel A represents Gender-by-race intersectional quadrants; Panel B is Gender-by-class; Panel C is Race-by-class. Occupations are colored according to their respective quadrant.

# **Study 1: Comparisons between occupational language stereotypes and ground-truth data**

Our primary analyses focus on documenting the relative frequencies of trait (and occupation) associations across intersectional quadrants to illustrate that some intersectional quadrants dominate language representations. As reported in the main text, comparisons between language-based stereotypes and ground-truth data for the overall frequencies of occupations in intersectional quadrants are statistically indistinguishable from one another. In other words, language and the real world are both capturing which intersectional groups dominate in the world versus which groups are made invisible and infrequent.

There are more granular comparisons that can also be computed, however. As discussed in the main text, we also explore the categorizations of individual occupations into the “correct” quadrants, based on their ground-truth classification. Figure S3 visualizes these cross-classifications. As can be seen below (and as reported in the main text) the cross-classifications show that, when considering the full 143 occupations, specific occupations were classified correctly around 40-50% of the time, and not significantly different from a 50-50 chance if treated as a binary classification task. For example, as seen in Figure S3, *cabinetmaker, pipelayer, logger*, and *agriculturalist,* are, in ground truth data, predominantly *White Men* occupations (they are in the top-right quadrant). Yet the language data had classified them as *Black Men* occupations (they are colored in light blue). Analogously, *butcher, driver*, and *chef* are, in ground truth data, relatively more *Black Men* (they are in the bottom-right quadrant); yet the language data had classified them as *White Men* occupations (they are colored in dark blue).

| **A.** | **** |
| --- | --- |
| **B.** | **** |
| **C.** | **** |
|  |  |

**Fig S3. Scatterplots of real-world demographic representation classifications of 143 occupations (z-scored).** Colors represent the classifications by language associations. If results of specific occupation classifications were identical between ground-truth and language associations, the colors of each quadrant would be consistent (e.g., all occupations in the top right quadrant would be colored dark blue). Panel A represents Gender-by-race intersectional quadrants; Panel B is Gender-by-class; Panel C is Race-by-class.

Such misclassifications could be seen as a limitation to the validity of the Flexible Intersectional Stereotype Extraction (FISE) approach for this setting. However, many of the misclassifications arise for those groups that provide relatively ambiguous signal (i.e., they sit around the 50% mark in gender representation). When we instead subset to investigate only those occupations that provide clear signal of their demographics in ground truth data, we find significant and above-chance accuracy. Specifically, we use stricter classification criteria as follows:

- For gender, <30% male is Female, >70% male is Male
- For race, <30 percentage point difference between White and Black is relatively Black, >70 percentage point difference between White and Black is relatively White
- For class, <$775 median weekly earnings is Poor (the bottom quartile), >$1250 median weekly earnings is Rich

We created lists of “extreme” occupations that conformed to those stricter classifications. The result was a list of 42 occupations that were classified as extreme in gender-by-race (Table S1), 33 occupations in gender-by-class (see R code), and 27 in race-by-class (see R code). Of those occupations, the hit rates improved substantially, to 29/42 correct for gender-by-race (69%), 23/33 (70%) for gender-by-class, and 19/27 (70%) for race-by-class. Full statistics are reported in the main text.

**Table S1.**

Extreme occupations in gender-by-race comparisons, with all “correct” occupations listed in standard font and no shading; the few “missed” occupations are listed in italicized font with shading.

| **Occupation** | **Classification from:** | | **Percent women** | **Percent white** | **Percent black** |
| --- | --- | --- | --- | --- | --- |
|  | **Language** | **BLS data** |  |  |  |
| **architect** | menwhite | menwhite | 29.8 | 87.7 | 4.6 |
| **broadcaster** | menwhite | menwhite | 6.3 | 82 | 8.5 |
| **carpenter** | menwhite | menwhite | 3.5 | 88.6 | 5.5 |
| **ceo** | menwhite | menwhite | 29.2 | 85.9 | 5.9 |
| **chiropractor** | menwhite | menwhite | 25.5 | 80.9 | 9 |
| **developer** | menwhite | menwhite | 21.9 | 80.2 | 0.8 |
| **drafter** | menwhite | menwhite | 15.3 | 88.2 | 5.1 |
| **electrician** | menwhite | menwhite | 2.2 | 88.5 | 7.3 |
| **firefighter** | menwhite | menwhite | 6.2 | 85.5 | 9.9 |
| **host** | womenwhite | womenwhite | 84 | 81.8 | 7.2 |
| **hygienist** | womenwhite | womenwhite | 96.3 | 91.4 | 2.5 |
| **inspector** | menwhite | menwhite | 11.8 | 85 | 9.7 |
| **installer** | menwhite | menwhite | 7.5 | 83.9 | 7.5 |
| **landscaper** | menwhite | menwhite | 7.2 | 85.2 | 7.1 |
| **machinist** | menwhite | menwhite | 6.8 | 88.2 | 5.7 |
| **manicurist** | womenblack | womenblack | 85.1 | 24.5 | 7.6 |
| **mechanic** | menwhite | menwhite | 4.2 | 85.7 | 8.5 |
| **painter** | menwhite | menwhite | 10.5 | 89.8 | 6.8 |
| **paramedic** | menwhite | menwhite | 23.6 | 90.9 | 1.6 |
| **pilot** | menwhite | menwhite | 9.2 | 95.7 | 2.6 |
| **plumber** | menwhite | menwhite | 1.1 | 83.3 | 8.9 |
| **psychologist** | womenwhite | womenwhite | 75 | 87.3 | 5.8 |
| **roofer** | menwhite | menwhite | 5 | 87.7 | 6.9 |
| **secretary** | womenwhite | womenwhite | 92.5 | 83.3 | 9.5 |
| **skincare** | womenwhite | womenwhite | 98.7 | 78.8 | 4.4 |
| **taper** | menwhite | menwhite | 2.8 | 92.8 | 0.8 |
| **taxidriver** | menblack | menblack | 13 | 50 | 26.3 |
| **teacher** | womenwhite | womenwhite | 79.7 | 82.5 | 11.1 |
| **welder** | menwhite | menwhite | 5.1 | 82.7 | 10.2 |
| ***agriculturalist*** | *menblack* | *menwhite* | *26.5* | *89.4* | *4.7* |
| ***bookkeeper*** | *womenblack* | *womenwhite* | *85* | *82.5* | *8.7* |
| ***cabinetmaker*** | *menblack* | *menwhite* | *10.3* | *97.3* | *2.2* |
| ***extractor*** | *womenwhite* | *menwhite* | *2.5* | *86.1* | *5* |
| ***fundraiser*** | *womenblack* | *womenwhite* | *82.9* | *91.8* | *0.3* |
| ***guard*** | *menwhite* | *menblack* | *24.3* | *54.9* | *34.5* |
| ***highwayman*** | *menblack* | *menwhite* | *3.9* | *83.4* | *12.9* |
| ***librarian*** | *womenblack* | *womenwhite* | *82.2* | *86* | *4.3* |
| ***logger*** | *menblack* | *menwhite* | *6.5* | *89.8* | *4.2* |
| ***pathologist*** | *menwhite* | *womenwhite* | *97.6* | *93.9* | *4* |
| ***pipelayer*** | *menblack* | *menwhite* | *2.4* | *97* | *2.3* |
| ***surveyer*** | *menblack* | *menwhite* | *11.3* | *89.2* | *8.5* |
| ***teller*** | *womenblack* | *womenwhite* | *83.1* | *79.8* | *9.4* |

We also performed another supplemental analysis to better understand the types of misclassification errors. Specifically, we examined whether errors are more likely to occur along single dimensions (e.g., within-genders, or within-race), rather than across both gender and race at once. We therefore recomputed the classifications of occupations but treated them as only binary classifications along gender (or along race, or class). For these simpler binary classification tasks, accuracy improved substantially, even across all 143 occupations (that included the ambiguous occupations). For gender-by-race, for example, binary classification along gender had significant and moderate accuracy of 78% [70%, 84%], p < .001, and binary classification along race had significant and moderate accuracy of 76% [67%, 82%], p < .001. In other words, very few errors occur where an occupation that was classified as relatedly *Men*-dominated in language is, in truth, relatively more *Women*-dominated. This result can also be seen visually in the fact that there is more misclassification among the light blue vs. dark blue (Figure S2, Panel A), and light green vs. dark green, than across blue vs. green.

# **Study 1: Comparisons between occupational language stereotypes and ground-truth data for individual group dimensions**

Although not strictly an intersectional analysis, we next can look to see if the language-based representations for a given singular dimension (e.g., gender) aligns with the ground-truth data for that dimension as well. Figure S4 visualizes these single dimension correlations. Results show that gender provides the clearest significant signal, with a correlation of *r* = .62 between language and real world data. Race, by contrast, provides the weakest signal *r* = -.017, although still in the direction we would expect, such that occupations that are more Black are also less likely to be associated with White (vs. Black) in language.

| **A.** | ** |
| --- | --- |
| **B.** | ** |
| **C.** | ** |
|  |  |

**Fig S4. Relationships of real-world demographic representation classifications of 143 occupations and language associations.** Panel A represents Gender dimension, showing a positive and significant correlation between occupation-men (vs. women) associations in language and percent men in the occupation; Panel B is Race, showing an expected negative and just-significant correlation between occupation-White (vs. Black) associations in language and percent Black in the occupation; Panel C is Class showing a positive correlation between occupation-Rich (vs. Poor) associations in language and median weekly earnings in the occupation.

# **Study 2: Comparisons to simpler (averaged vector) approach**

A simpler approach to investigating intersectional stereotypes would be to average across combined lists of words for multiple group identities all at once. To illustrate the difference between FISE and an averaged vector approach, imagine an example of looking at race-by-gender intersectional stereotypes. To recap: in the FISE approach, we would first project all traits along a gender axis defined by the relative association of each trait to 12 men words versus 12 women words. Then we project all traits along a second, race axis defined, in this case, by the relative association of each trait to 12 White words versus 12 Black words. Finally, we cross those two axes to create an *x-y* space and see which traits fall in each of the quadrants.

In contrast, in the simple averaged vector approach, we create four lists of combined group words, one for each of the four intersectional groupings – *White+men, White+women, Black+men,* and *Black+women*. Each of these four lists comprises 24 words, with 12 words referring to the gender group and 12 referring to the racial group. Then, we use mean average cosine (MAC; Manzini et al., 2019; Charlesworth, Caliskan, & Banaji, 2022) to examine the degree of association between each trait and the combined list of intersectional group words. Higher positive associations indicate that those traits are more associated with the intersectional group, while negative associations indicate that those traits are inversely associated to the intersectional group. Finally, for comparing the outputs between FISE and simple averaged vector approach, we take only the top-25 traits associated with the averaged intersectional group vector.

There are a few key differences between FISE and the simple averaged vector approach. First, whereas FISE computes all associations *relatively* (i.e., the relative association to men versus women), the simple averaged vector approach computes absolute associations to the combined lists of words. Absolute associations have the disadvantage of including any words that may describe all social groups, in general, rather than words that describe the unique *stereotypical* associations of just one intersectional group.

A second key difference is that FISE computes the associations with constituent (parent) groups (e.g., White, men) separately, whereas the simple averaged vector approach computes the associations to the intersection all at once. Computing separate associations to constituent groups helps reveal *why* a trait may be associated with an intersection. For example, the simple averaged vector approach reveals that the trait *arrogant* is associated with the intersection of *White+Rich,* but it cannot tell us whether that trait is equally associated with Whiteness and Richness, more associated with Whiteness than Richness, or vice versa. In short, the simple averaged vector approach limits our understanding of how intersectional traits are “inherited” from their parent constituent groups.

These key differences in methodologies contribute to parallel differences in results as well (Table S2). For example, in FISE, the *relative* nature of the analyses pulls out the finding that Rich, White, and men are generally more associated with positive traits, while Poor, Black, and women are generally more associated with negative traits. However, using the *absolute* results from the simple averaged vectors, we find that, in general, most groups are associated with similar traits and, moreover, that most of those top-associated traits are negative in valence. For instance, 80% of traits associated with *White+Rich* are negative, and 96% of traits associated with *White+Poor* are negative. The traits *helpless, greedy,* and *heartless,* for example, are also associated with both groups. Such findings emphasize the usefulness of FISE as a new approach to uncover the unique and specific stereotypic associations with intersectional groups, rather than group-general concepts that arise from a simple averaging approach.

**Table S2.**

Relative proportions of traits (by valence, arousal, dominance, warmth, and competence) within the top 25 traits associated with the averaged intersectional group for GloVe 840B embeddings

|  | **Valence** | | **Warmth** | | **Competence** | | **Arousal** | | **Dominance** | |
| --- | --- | --- | --- | --- | --- | --- | --- | --- | --- | --- |
| **Intersectional grouping** | **Positive** | **Negative** | **Warm** | **Cold** | **Comp** | **Incomp** | **High** | **Low** | **High** | **Low** |
| **White + Rich** | 20 | 80 | 24 | 76 | 20 | 80 | 52 | 48 | 72 | 28 |
| **White + Poor** | 4 | 96 | 4 | 96 | 4 | 96 | 56 | 44 | 88 | 12 |
| **Black + Rich** | 24 | 76 | 28 | 72 | 28 | 72 | 52 | 48 | 64 | 36 |
| **Black + Poor** | 4 | 96 | 4 | 96 | 4 | 96 | 60 | 40 | 84 | 16 |
| **Male + Rich** | 48 | 52 | 48 | 52 | 48 | 52 | 52 | 48 | 48 | 52 |
| **Male + Poor** | 20 | 80 | 20 | 80 | 20 | 80 | 52 | 48 | 72 | 28 |
| **Female + Rich** | 56 | 44 | 56 | 44 | 56 | 44 | 52 | 48 | 40 | 60 |
| **Female + Poor** | 24 | 76 | 24 | 76 | 24 | 76 | 48 | 52 | 68 | 32 |
| **Male + White** | 44 | 56 | 44 | 56 | 44 | 56 | 52 | 48 | 48 | 52 |
| **Male + Black** | 36 | 64 | 36 | 64 | 36 | 64 | 48 | 52 | 60 | 40 |
| **Female + White** | 36 | 64 | 36 | 64 | 36 | 64 | 56 | 44 | 56 | 44 |
| **Female + Black** | 40 | 60 | 40 | 60 | 40 | 60 | 48 | 52 | 56 | 44 |

*Note.* Each number reflects the percentage of traits within the top-25 traits (listed in Table S3) that are, for example, coded as positive (vs. negative).

**Table S3.**

Top 25 traits associated with the intersectional group, based on simple averaged vectors combining across group lists.

| **Intersectional grouping** | **Top-25 associated traits** |
| --- | --- |
| **White + Rich** | *arrogant, greedy, prejudiced, immoral, intellectual, conceited, heartless, discriminating, cowardly, jealous, fearful, stupid, hostile, brave, irresponsible, insecure, intelligent, loyal, incompetent, dishonest, cruel, obnoxious, helpless, carefree, angry* |
| **White + Poor** | *helpless, lonely, greedy, depressed, heartless, insecure, arrogant, cowardly, stupid, prejudiced, immoral, fearful, angry, incompetent, rude, brave, cruel, irresponsible, conceited, insolent, disrespectful, inconsiderate, unfriendly, jealous, dishonest* |
| **Black + Rich** | *greedy, arrogant, prejudiced, immoral, conceited, fearful, insolent, heartless, discriminating, cowardly, intellectual, helpless, cruel, dishonest, hostile, scornful, insecure, jealous, loyal, brave, courageous, intelligent, incompetent, spirited, stingy* |
| **Black + Poor** | *helpless, lonely, prejudiced, immoral, fearful, insolent, greedy, insecure, heartless, depressed, cowardly, cruel, conceited, angry, arrogant, disrespectful, incompetent, brave, lifeless, stingy, dishonest, stupid, scornful, irresponsible, deceitful* |
| **Male + Rich** | *arrogant, jealous, greedy, brave, loyal, kind, angry, cruel, lonely, happy, honest, intellectual, gracious, courageous, helpless, fearful, heartless, affectionate, talented, immoral, stupid, generous, cowardly, obnoxious, spirited* |
| **Male + Poor** | *lonely, helpless, brave, angry, jealous, cruel, depressed, greedy, arrogant, fearful, happy, kind, stupid, heartless, grumpy, abusive, rude, courageous, cowardly, incompetent, honest, insecure, cranky, obnoxious, irresponsible* |
| **Female + Rich** | *jealous, lonely, greedy, brave, arrogant, cruel, kind, angry, helpless, loyal, happy, spirited, gracious, fearful, courageous, romantic, talented, affectionate, heartless, sexy, abusive, honest, immoral, intellectual, feminine* |
| **Female + Poor** | *lonely, helpless, jealous, angry, brave, cruel, depressed, fearful, abusive, happy, insecure, heartless, greedy, kind, cranky, rude, courageous, grumpy, stupid, irresponsible, obnoxious, immoral, cheerful, hopeful, arrogant* |
| **Male + White** | *jealous, stupid, brave, angry, lonely, arrogant, happy, rude, kind, cruel, sexy, honest, jolly, greedy, grumpy, affectionate, helpless, obnoxious, cowardly, disrespectful, cheerful, courageous, heartless, smart, loyal* |
| **Male + Black** | *angry, jealous, brave, lonely, stupid, sexy, cruel, arrogant, rude, helpless, happy, kind, courageous, affectionate, honest, disrespectful, greedy, insolent, cowardly, prejudiced, grumpy, obnoxious, jolly, fearful, loyal* |
| **Female + White** | *jealous, sexy, lonely, angry, stupid, brave, happy, rude, cruel, kind, helpless, arrogant, bossy, feminine, affectionate, cheerful, grumpy, romantic, honest, obnoxious, disrespectful, greedy, heartless, depressed, fearful* |
| **Female + Black** | *sexy, jealous, lonely, angry, brave, helpless, cruel, stupid, feminine, rude, bossy, happy, affectionate, courageous, sensual, disrespectful, kind, fearful, spirited, arrogant, insolent, cheerful, prejudiced, heartless, obnoxious* |

*Note.* Traits are ordered according to their strength of association, with the most-associated traits listed first.

# **Study 2: Illustration of three-way race-by-gender-by-class intersectional analyses**

We intentionally named FISE as the *Flexible* Intersectional Stereotype Extraction approach to emphasize the possibility of generalizing this procedure across many settings. In particular, one obvious direction for generalization is to move beyond the most typical two-way intersections (those that are most frequent in perception and, correspondingly, also in research; Petsko & Bodenhausen, 2022). Here, we therefore illustrate an application of FISE to a three-way analysis in which we simultaneously cross gender, race, and class.

Results illustrated in Tables S4 and S5 and in Figure S5 reinforce the dominance of White, Rich, men in large-scale English language embedding spaces. For instance, similar to the results for two-way analyses, we find that 41% of all possible traits are associated with *White+Rich+Men,* whereas only 2% are associated with *Black+Rich+Women.* Moreover, even after re-equated with z-scoring, the results continue to show the obvious imbalances in the features of traits associated with each intersectional quadrant: 78% of the traits associated with *White+Rich+Men* are positive, whereas 85% of the traits associated with *Black+Poor+Women* are negative.

**Table S4.**

Overall frequencies (from raw associations) and relative proportions of features of traits (from z-scored) defined by the three-way intersectional spaces of gender, race, and class

| **Intersectional grouping** |  | **Valence** | | **Warmth** | | **Competence** | | **Arousal** | | **Dominance** | |
| --- | --- | --- | --- | --- | --- | --- | --- | --- | --- | --- | --- |
|  | **Overall Freq.** | **Positive** | **Negative** | **Warm** | **Cold** | **Comp** | **Incomp** | **High** | **Low** | **High** | **Low** |
| **Men+White+Rich** | 41 | 78 | 22 | 56 | 44 | 78 | 22 | 22 | 78 | 78 | 22 |
| **Men+White+Poor** | 18 | 29 | 71 | 36 | 64 | 29 | 71 | 71 | 29 | 29 | 71 |
| **Men+Black+Rich** | 3 | 58 | 42 | 50 | 50 | 58 | 42 | 42 | 58 | 58 | 42 |
| **Men+Black+Poor** | 3 | 27 | 73 | 55 | 45 | 27 | 73 | 73 | 27 | 27 | 73 |
| **Women+White+Rich** | 16 | 78 | 22 | 44 | 56 | 78 | 22 | 11 | 89 | 78 | 22 |
| **Women+White+Poor** | 14 | 22 | 78 | 78 | 22 | 22 | 78 | 78 | 22 | 22 | 78 |
| **Women+Black+Rich** | 2 | 79 | 21 | 57 | 43 | 71 | 29 | 21 | 79 | 79 | 21 |
| **Women+Black+Poor** | 3 | 15 | 85 | 31 | 69 | 15 | 85 | 85 | 15 | 23 | 77 |

**Table S5**

Traits falling in each of the eight quadrants defined by the three-way intersectional spaces of gender, race, and class

| **Intersectional grouping** | **Associated traits** |
| --- | --- |
| **Men+White+Rich** | *accurate, adventurous, affectionate, arrogant, brilliant, clever, conceited, confident, dependable, discriminating, dishonest, energetic, enthusiastic, friendly, generous, greedy, happy, helpful, honest, hostile, humorous, intellectual, intelligent, jolly, kind, knowledgeable, loyal, obnoxious, optimistic, original, peaceful, pleasant, relaxed, reliable, smart, thoughtful, trustworthy, truthful, unethical, wasteful, witty* |
| **Men+White+Poor** | *angry, brave, charitable, cowardly, cruel, disrespectful, grim, grumpy, heartless, incompetent, inconsiderate, irresponsible, negligent, rude, stingy, stupid, unreliable, weak* |
| **Men+Black+Rich** | *courageous, immoral, prejudiced* |
| **Men+Black+Poor** | *deceitful, disorderly, insolent* |
| **Women+White+Rich** | *bossy, carefree, compassionate, feminine, gentle, gracious, jealous, manipulative, playful, resourceful, romantic, sexy, spirited, talented, tense, warm* |
| **Women+White+Poor** | *abusive, cheerful, cranky, depressed, fearful, fussy, glum, hopeful, insecure, irritable, lonely, spiteful, unfriendly, unkind* |
| **Women+Black+Rich** | *scornful, sensual* |
| **Women+Black+Poor** | *helpless, lifeless, uncooperative* |

**Fig S5. Three-way intersectional trait associations with gender (x-axis), class (y-axis) and race (z-axis).** Traits are colored according to which quadrant they fall into. Dashed lines indicate the zero coordinates for each axis.

# **Study 2: Illustration of intersectional analyses for French embeddings**

As above, the *flexibility* of FISE is, we argue, its key contribution to the literature and methods of intersectional analyses. In addition to the flexibility described above, FISE can theoretically be applied across any non-English language as well. Here, we therefore summarize the results from an application of FISE to French embeddings, obtained from *fastText* embeddings trained on French Wikipedia and Common Crawl text. In addition, the accompanying streamlined R code (provided in the project’s OSF page) can be used to “slot in” whichever group terms and embedding space the user may want to study.

We cannot replicate all analyses and results in French as we would need similar word norms (i.e., ratings of the French traits along valence, arousal, dominance, warmth, and competence, as in the English analyses). Nevertheless, we can illustrate the broad-stroke conclusions that are similar even in the French setting. As shown in Table S6, French embeddings also show androcentric and ethnocentric dominance in language frequencies: *White+Men*, for example, are associated with 52% of traits, while *Black+Women* are associated with only 2% of traits.

Additionally, as shown in Figure S6, the descriptive patterns of positive versus negative traits appear to follow what was seen in the primary English analyses: of the traits most associated with *Black+Men*, for example, we see mostly negative content, including *cruel* (tr. cruel)*, immoral* (tr. immoral)*, impoli* (tr. impolite)*, odieux* (tr. odious)*, gaspilleur* (tr. wasteful)*;* of the traits associated with *White+Men,* by contrast, we see more positive content, including *compétent* (tr. competent)*, attentionné* (tr. attentive)*, original* (tr. original)*, confiant* (tr. confident)*, détendu* (tr. relaxed)*.*

**Table S6.**

Overall frequencies (from raw associations) for French embeddings

| **Intersectional grouping** | **Overall freq.** |
| --- | --- |
| **White + Rich** | 36 |
| **White + Poor** | 29 |
| **Black + Rich** | 5 |
| **Black + Poor** | 30 |
| **Male + Rich** | 32 |
| **Male + Poor** | 53 |
| **Female + Rich** | 9 |
| **Female + Poor** | 6 |
| **Male + White** | 52 |
| **Male + Black** | 33 |
| **Female + White** | 13 |
| **Female + Black** | 2 |

| ***A.*** | ****** |
| --- | --- |
| ***B.*** | ****** |
| ***C.*** | ****** |

***Fig S6.* French intersectional trait associations for intersections of (A) gender-by-race, (B) gender-by-class, and (C) race-by-class.** Colors represent the classifications of traits into intersectional quadrants by their relative associations with the groupings in language.

# **Study 2: Relative features of traits for all embedding methods**

In the main text, we report the results for features of trait valence, warmth, competence, dominance, arousal, and word commonality for one embedding algorithm and dataset (*GloVe 840B*). In this section we report the parallel results for all other embedding methods/datasets. The primary analysis of the relative features of traits exhibits general consistency across all embedding models. Traits associated with dominant groups, such as *Rich, White, or Men*, were characterized by positivity, warmth, competence, and dominance. Again, notably, class played a significant role in shaping the features associated with groups, such that high class (Rich) groups consistently exhibited "halos" in terms of trait features.

***Table S7.***

Relative percentages of types of traits (by valence, warmth and competence) within each intersectional quadrant for **GloVe Wikipedia 6B static embeddings.**

|  | **Valence** | | **Warmth** | | **Competence** | | **Word Arousal** | | **Word Dominance** | |
| --- | --- | --- | --- | --- | --- | --- | --- | --- | --- | --- |
| **Intersectional grouping** | **Positive** | **Negative** | **Warm** | **Cold** | **Comp** | **Incomp** | **High** | **Low** | **High** | **Low** |
| **White + Rich** | 76 | 24 | 76 | 24 | 76 | 24 | 47 | 53 | 76 | 24 |
| **White + Poor** | 13 | 87 | 13 | 87 | 13 | 87 | 47 | 53 | 13 | 87 |
| **Black + Rich** | 72 | 28 | 78 | 22 | 78 | 22 | 50 | 50 | 67 | 33 |
| **Black + Poor** | 27 | 73 | 27 | 73 | 27 | 73 | 55 | 45 | 27 | 73 |
| **Male + Rich** | 78 | 22 | 78 | 22 | 81 | 19 | 50 | 50 | 78 | 22 |
| **Male + Poor** | 30 | 70 | 30 | 70 | 30 | 70 | 55 | 45 | 30 | 70 |
| **Female + Rich** | 70 | 30 | 75 | 25 | 70 | 30 | 45 | 55 | 65 | 35 |
| **Female + Poor** | 18 | 82 | 18 | 82 | 18 | 82 | 50 | 50 | 18 | 82 |
| **Male + White** | 68 | 32 | 68 | 32 | 71 | 29 | 58 | 42 | 68 | 32 |
| **Male + Black** | 48 | 52 | 48 | 52 | 48 | 52 | 43 | 57 | 48 | 52 |
| **Female + White** | 39 | 61 | 39 | 61 | 33 | 67 | 28 | 72 | 39 | 61 |
| **Female + Black** | 40 | 60 | 43 | 57 | 43 | 56 | 60 | 40 | 37 | 63 |

*Note.* Each number reflects the percentage of traits from the given quadrant (e.g., from the intersectional grouping of *White+Rich*) that are, for example, coded as positive (vs. negative).

***Table S8.***

Relative percentages of types of traits (by valence, warmth and competence) within each intersectional quadrant for **fastText Wikipedia 2M static embeddings.**

|  | **Valence** | | **Warmth** | | **Competence** | | **Word Arousal** | | **Word Dominance** | |
| --- | --- | --- | --- | --- | --- | --- | --- | --- | --- | --- |
| **Intersectional grouping** | **Positive** | **Negative** | **Warm** | **Cold** | **Comp** | **Incomp** | **High** | **Low** | **High** | **Low** |
| **White + Rich** | 84 | 16 | 84 | 16 | 81 | 19 | 52 | 48 | 84 | 16 |
| **White + Poor** | 40 | 60 | 40 | 60 | 40 | 60 | 35 | 65 | 40 | 60 |
| **Black + Rich** | 58 | 42 | 63 | 37 | 63 | 37 | 53 | 47 | 53 | 47 |
| **Black + Poor** | 17 | 83 | 17 | 83 | 20 | 80 | 57 | 43 | 17 | 83 |
| **Male + Rich** | 74 | 26 | 74 | 26 | 74 | 26 | 52 | 48 | 74 | 26 |
| **Male + Poor** | 32 | 68 | 32 | 68 | 32 | 68 | 48 | 52 | 32 | 68 |
| **Female + Rich** | 74 | 26 | 78 | 22 | 74 | 26 | 52 | 48 | 70 | 30 |
| **Female + Poor** | 20 | 80 | 20 | 80 | 24 | 76 | 48 | 52 | 20 | 80 |
| **Male + White** | 69 | 31 | 69 | 31 | 69 | 31 | 47 | 53 | 69 | 31 |
| **Male + Black** | 30 | 70 | 30 | 70 | 30 | 70 | 55 | 45 | 30 | 70 |
| **Female + White** | 63 | 37 | 63 | 37 | 58 | 42 | 42 | 58 | 63 | 37 |
| **Female + Black** | 34 | 66 | 38 | 62 | 41 | 59 | 55 | 45 | 31 | 69 |

*Note.* Each number reflects the percentage of traits from the given quadrant (e.g., from the intersectional grouping of *White+Rich*) that are, for example, coded as positive (vs. negative).

***Table S9.***

Relative percentages of types of traits (by valence, warmth and competence) within each intersectional quadrant for **BERT embeddings extracted as single words with templates.**

|  | **Valence** | | **Warmth** | | **Competence** | | **Word Arousal** | | **Word Dominance** | |
| --- | --- | --- | --- | --- | --- | --- | --- | --- | --- | --- |
| **Intersectional grouping** | **Positive** | **Negative** | **Warm** | **Cold** | **Comp** | **Incomp** | **High** | **Low** | **High** | **Low** |
| **White + Rich** | 80 | 20 | 80 | 20 | 78 | 22 | 43 | 57 | 80 | 20 |
| **White + Poor** | 36 | 64 | 36 | 64 | 43 | 57 | 57 | 43 | 36 | 64 |
| **Black + Rich** | 71 | 29 | 79 | 21 | 79 | 21 | 50 | 50 | 64 | 36 |
| **Black + Poor** | 9 | 91 | 9 | 91 | 9 | 91 | 53 | 47 | 9 | 91 |
| **Male + Rich** | 83 | 17 | 86 | 14 | 86 | 14 | 45 | 55 | 83 | 17 |
| **Male + Poor** | 9 | 91 | 9 | 91 | 13 | 87 | 56 | 44 | 9 | 91 |
| **Female + Rich** | 72 | 28 | 72 | 28 | 68 | 32 | 44 | 56 | 68 | 32 |
| **Female + Poor** | 26 | 74 | 26 | 74 | 26 | 74 | 52 | 48 | 26 | 74 |
| **Male + White** | 69 | 31 | 69 | 31 | 72 | 28 | 48 | 52 | 69 | 31 |
| **Male + Black** | 26 | 74 | 30 | 70 | 30 | 70 | 52 | 48 | 26 | 74 |
| **Female + White** | 68 | 32 | 68 | 32 | 64 | 36 | 44 | 56 | 68 | 32 |
| **Female + Black** | 30 | 70 | 30 | 70 | 30 | 70 | 52 | 48 | 26 | 74 |

*Note.* Each number reflects the percentage of traits from the given quadrant (e.g., from the intersectional grouping of *White+Rich*) that are, for example, coded as positive (vs. negative).

***Table S10.***

Relative percentages of types of traits (by valence, warmth and competence) within each intersectional quadrant for **BERT embeddings, pooled words with template.**

|  | **Valence** | | **Warmth** | | **Competence** | | **Word Arousal** | | **Word Dominance** | |
| --- | --- | --- | --- | --- | --- | --- | --- | --- | --- | --- |
| **Intersectional grouping** | **Positive** | **Negative** | **Warm** | **Cold** | **Comp** | **Incomp** | **High** | **Low** | **High** | **Low** |
| **White + Rich** | 77 | 23 | 77 | 23 | 79 | 21 | 54 | 46 | 77 | 23 |
| **White + Poor** | 0 | 100 | 0 | 100 | 0 | 100 | 50 | 50 | 0 | 100 |
| **Black + Rich** | 83 | 17 | 83 | 17 | 83 | 17 | 33 | 67 | 67 | 33 |
| **Black + Poor** | 14 | 86 | 17 | 83 | 14 | 86 | 45 | 55 | 14 | 86 |
| **Male + Rich** | 82 | 18 | 82 | 18 | 85 | 15 | 42 | 58 | 82 | 18 |
| **Male + Poor** | 21 | 79 | 26 | 74 | 26 | 74 | 58 | 42 | 21 | 79 |
| **Female + Rich** | 72 | 28 | 72 | 28 | 72 | 28 | 64 | 36 | 68 | 32 |
| **Female + Poor** | 4 | 96 | 4 | 96 | 0 | 100 | 35 | 65 | 4 | 96 |
| **Male + White** | 73 | 27 | 73 | 27 | 76 | 24 | 45 | 55 | 73 | 27 |
| **Male + Black** | 37 | 63 | 42 | 58 | 42 | 58 | 53 | 47 | 37 | 63 |
| **Female + White** | 64 | 36 | 64 | 36 | 64 | 36 | 64 | 36 | 64 | 36 |
| **Female + Black** | 13 | 87 | 13 | 87 | 9 | 91 | 35 | 65 | 9 | 91 |

*Note.* Each number reflects the percentage of traits from the given quadrant (e.g., from the intersectional grouping of *White+Rich*) that are, for example, coded as positive (vs. negative).

***Table S11.***

Relative percentages of types of traits (by valence, warmth and competence) within each intersectional quadrant for **BERT embeddings, pooled words with no template.**

|  | **Valence** | | **Warmth** | | **Competence** | | **Word Arousal** | | **Word Dominance** | |
| --- | --- | --- | --- | --- | --- | --- | --- | --- | --- | --- |
| **Intersectional grouping** | **Positive** | **Negative** | **Warm** | **Cold** | **Comp** | **Incomp** | **High** | **Low** | **High** | **Low** |
| **White + Rich** | 82 | 18 | 82 | 18 | 82 | 18 | 52 | 48 | 79 | 21 |
| **White + Poor** | 29 | 71 | 29 | 71 | 36 | 64 | 50 | 50 | 29 | 71 |
| **Black + Rich** | 72 | 28 | 72 | 28 | 72 | 28 | 56 | 44 | 72 | 28 |
| **Black + Poor** | 17 | 83 | 20 | 80 | 17 | 82 | 43 | 57 | 17 | 83 |
| **Male + Rich** | 69 | 31 | 69 | 31 | 69 | 31 | 46 | 54 | 69 | 31 |
| **Male + Poor** | 15 | 85 | 19 | 81 | 22 | 78 | 56 | 44 | 15 | 85 |
| **Female + Rich** | 88 | 12 | 88 | 12 | 88 | 12 | 60 | 40 | 84 | 16 |
| **Female + Poor** | 27 | 73 | 27 | 73 | 23 | 77 | 32 | 68 | 27 | 73 |
| **Male + White** | 56 | 44 | 56 | 44 | 60 | 40 | 48 | 52 | 56 | 44 |
| **Male + Black** | 29 | 71 | 32 | 68 | 32 | 68 | 54 | 46 | 29 | 71 |
| **Female + White** | 77 | 23 | 77 | 23 | 77 | 23 | 55 | 45 | 73 | 27 |
| **Female + Black** | 44 | 56 | 44 | 56 | 40 | 60 | 40 | 60 | 44 | 56 |

*Note.* Each number reflects the percentage of traits from the given quadrant (e.g., from the intersectional grouping of *White+Rich*) that are, for example, coded as positive (vs. negative).

***Table S12.***

Commonality of traits (Zipf scores) in everyday language for each intersectional quadrant, by embedding method (from z-scored results)

|  | ***Static embeddings*** | | | ***Contextualized embeddings*** | | |
| --- | --- | --- | --- | --- | --- | --- |
| ***Intersectional grouping*** | ***GloVe CC 840B*** | ***GloVe Wiki 6B*** | ***fastText Wiki 2M*** | ***Single words with template*** | ***Pooled words with template*** | ***Pooled words no template*** |
| **White + Rich** | 4.13 | 4.16 | 4.10 | 3.89 | 4.16 | 4.15 |
| **White + Poor** | 3.88 | 4.04 | 3.75 | 3.73 | 3.91 | 3.74 |
| **Black + Rich** | 3.56 | 3.44 | 3.46 | 3.62 | 3.46 | 4.00 |
| **Black + Poor** | 3.41 | 3.35 | 3.61 | 3.65 | 3.19 | 3.26 |
| **Male + Rich** | 4.01 | 4.05 | 3.88 | 3.80 | 4.05 | 4.07 |
| **Male + Poor** | 3.78 | 3.85 | 3.78 | 3.51 | 3.31 | 3.39 |
| **Female + Rich** | 3.63 | 3.73 | 3.80 | 3.85 | 4.13 | 4.13 |
| **Female + Poor** | 3.48 | 3.36 | 3.54 | 3.84 | 3.29 | 3.41 |
| **Male + White** | 4.15 | 4.18 | 4.02 | 3.79 | 4.08 | 3.99 |
| **Male + Black** | 3.57 | 3.59 | 3.62 | 3.51 | 3.26 | 3.48 |
| **Female + White** | 3.77 | 4.01 | 3.92 | 3.91 | 4.20 | 4.07 |
| **Female + Black** | 3.42 | 3.26 | 3.50 | 3.77 | 3.21 | 3.54 |

***Note.*** Results reflect the commonality of traits in each quadrant for each embedding method (e.g., race-by-class). The commonality values are reported in zipf form, a phenomenon commonly observed in natural language, where the frequency of words follows a power-law distribution. A word's Zipf frequency is the base-10 logarithm of the number of times it appears in a billion words. For instance, the zipf value of 4.14 for the *White+Rich* quadrant reflects that the traits associated with this quadrant have occurred in language 10^4.14 or approximately 13803 times for every 1 billion words.

# **Study 2: Robustness across variations in number of traits**

As outlined across the next few sections, the key findings remain robust across variations in the precise model specifications, including the number of traits, the exact words used to represent groups, and whether the target list of concepts are trait adjectives, nouns, or verbs. First, irrespective of the number of traits used (100, 200, 300), patterns of andro-, ethno-, and, to a lesser extent, class-centrism persisted in raw frequencies. Moreover, even after mathematically aligning frequencies using z-scoring, we observed differences in the features of traits across intersectional groupings, with relatively greater positivity, warmth, competence, and dominance for intersectional quadrants including *White, Rich, or Men*.

***Table S13.***

Overall percentage of traits, **from list of 200 traits**, in each intersectional grouping (quadrant) by embedding method (non z-scored).

|  | ***Static embeddings*** | | | ***Contextualized embeddings*** | | |
| --- | --- | --- | --- | --- | --- | --- |
| ***Intersectional grouping*** | ***GloVe CC 840B*** | ***GloVe Wiki 6B*** | ***fastText Wiki 2M*** | ***Single words with template*** | ***Pooled words with template*** | ***Pooled words no template*** |
| **White + Rich** | **60.5** | **44.5** | **35.5** | **60.5** | **71.0** | **49.0** |
| **White + Poor** | 30.0 | 23.5 | 30.0 | 28.0 | 20.5 | 24.5 |
| **Black + Rich** | 3.5 | 9.5 | 9.5 | 1.0 | 0 | 3.0 |
| **Black + Poor** | 6.0 | 22.5 | 25.0 | 10.5 | 8.5 | 23.5 |
| **Male + Rich** | **44.5** | **35.0** | 24.0 | 20.0 | 30.5 | 20.0 |
| **Male + Poor** | 21.0 | 22.5 | **33.0** | 15.0 | 7.5 | 18.5 |
| **Female + Rich** | 19.5 | 19.0 | 21.0 | **41.5** | **40.5** | **32.0** |
| **Female + Poor** | 15.0 | 23.5 | 22.0 | 23.5 | 21.5 | 29.5 |
| **Male + White** | **60.0** | **46.5** | **40.0** | 28.0 | 36.5 | 28.0 |
| **Male + Black** | 5.5 | 11.0 | 17.0 | 7.0 | 1.5 | 10.5 |
| **Female + White** | 30.5 | 21.5 | 25.5 | **60.5** | **55.0** | **45.5** |
| **Female + Black** | 4.0 | 21.0 | 17.5 | 4.5 | 7.0 | 16.0 |

***Note.*** Trait frequencies represent the percentage of traits (in this case out of 200 possible traits) that are associated with each intersectional grouping (e.g., the *Male+White* grouping is associated with 60% of the 200 traits, whereas the *Male+Black* grouping is associated with 5.5% of the 200 traits). Frequencies are compared within a data source (e.g., *GloVe CC 840B*) but across groupings, such that all four groupings within a data source will add up to 100. Bolded numbers indicate the highest relative percentage for each intersectional quadrant (e.g., 60% is bolded to reflect that *Male+White* is the grouping with the highest relative percentage of traits across all groupings for that data source). The three contextualized embedding columns indicate three methods for extracting embedding vectors for groups. As described in the main text*, single words with templates* indicates that a vector is created from averaging across the hidden state vectors for the template (e.g., “This is a”) and the first sub-tokens of group words (e.g., “rich African woman”); *pooled words with templates* indicates that the vector is created from averaging across the hidden state vectors for the template and the pooled group words (pooled across sub-tokens; e.g., “rich African+American woman); pooled words no template indicates that the vector is created from the first four layers of the hidden state vectors pooled across only the group words (e.g., rich African+American woman).

***Table S14.***

Overall percentage of traits, **from list of 300 traits**, in each intersectional grouping (quadrant) by embedding method (non z-scored)

|  | ***Static embeddings*** | | | ***Contextualized embeddings*** | | |
| --- | --- | --- | --- | --- | --- | --- |
| ***Intersectional grouping*** | ***GloVe CC 840B*** | ***GloVe Wiki 6B*** | ***fastText Wiki 2M*** | ***Single words with template*** | ***Pooled words with template*** | ***Pooled words no template*** |
| **White + Rich** | **63.0** | **46.0** | **40.3** | **62.7** | **72.0** | **50.0** |
| **White + Poor** | 27.0 | 22.0 | 27.7 | 26.7 | 20.0 | 24.7 |
| **Black + Rich** | 4.3 | 11.3 | 11.7 | 0.7 | 0 | 3.3 |
| **Black + Poor** | 5.7 | 20.7 | 20.3 | 10 | 8.0 | 22.0 |
| **Male + Rich** | **47.3** | **37.3** | **31.3** | 19.3 | 30.0 | 18.7 |
| **Male + Poor** | 18.3 | 19.7 | 26.0 | 15.0 | 8.3 | 17.3 |
| **Female + Rich** | 20.0 | 20.0 | 20.7 | **44.0** | **42.0** | **34.7** |
| **Female + Poor** | 14.3 | 23.0 | 22.0 | 21.7 | 19.7 | 29.3 |
| **Male + White** | **60.3** | **46.7** | **40.7** | 28.3 | 36.3 | 25.3 |
| **Male + Black** | 5.3 | 10.3 | 16.7 | 6.0 | 2.0 | 10.7 |
| **Female + White** | 29.7 | 21.3 | 27.3 | **61.0** | **55.7** | **49.3** |
| **Female + Black** | 4.7 | 21.7 | 15.3 | 4.7 | 6.0 | 14.7 |

***Note.*** See note to Table S8. In this case, raw frequencies are divided by 3 to make them comparable to the 100 trait lists. Each number reflects the percent of the top 300 positive/negative traits associated with each intersectional quadrant.

***Table S15.***

Relative proportions of types of traits (by valence, warmth and competence) within each intersectional quadrant for **GloVe 840B embeddings, with 200 traits**

|  | **Valence** | | **Warmth** | | **Competence** | | **Word Arousal** | | **Word Dominance** | |
| --- | --- | --- | --- | --- | --- | --- | --- | --- | --- | --- |
| **Intersectional grouping** | **Positive** | **Negative** | **Warm** | **Cold** | **Comp** | **Incomp** | **High** | **Low** | **High** | **Low** |
| **White + Rich** | 0.77 | 0.23 | 0.79 | 0.21 | 0.81 | 0.19 | 0.44 | 0.56 | 0.79 | 0.21 |
| **White + Poor** | 0.26 | 0.74 | 0.26 | 0.74 | 0.28 | 0.72 | 0.42 | 0.58 | 0.26 | 0.74 |
| **Black + Rich** | 0.75 | 0.25 | 0.75 | 0.25 | 0.75 | 0.25 | 0.50 | 0.50 | 0.73 | 0.27 |
| **Black + Poor** | 0.17 | 0.83 | 0.17 | 0.83 | 0.19 | 0.81 | 0.60 | 0.40 | 0.17 | 0.83 |
| **Male + Rich** | 0.76 | 0.24 | 0.76 | 0.24 | 0.77 | 0.23 | 0.37 | 0.63 | 0.77 | 0.23 |
| **Male + Poor** | 0.27 | 0.73 | 0.27 | 0.73 | 0.30 | 0.70 | 0.52 | 0.48 | 0.30 | 0.70 |
| **Female + Rich** | 0.77 | 0.23 | 0.79 | 0.21 | 0.79 | 0.21 | 0.60 | 0.40 | 0.74 | 0.26 |
| **Female + Poor** | 0.16 | 0.84 | 0.16 | 0.84 | 0.18 | 0.82 | 0.51 | 0.49 | 0.14 | 0.86 |
| **Male + White** | 0.57 | 0.43 | 0.57 | 0.43 | 0.60 | 0.40 | 0.43 | 0.57 | 0.60 | 0.40 |
| **Male + Black** | 0.54 | 0.46 | 0.54 | 0.46 | 0.54 | 0.46 | 0.44 | 0.56 | 0.54 | 0.46 |
| **Female + White** | 0.51 | 0.49 | 0.54 | 0.46 | 0.54 | 0.46 | 0.43 | 0.57 | 0.49 | 0.51 |
| **Female + Black** | 0.39 | 0.61 | 0.39 | 0.61 | 0.41 | 0.59 | 0.63 | 0.37 | 0.37 | 0.63 |

*Note.* Each number reflects the percentage of traits from the given quadrant (e.g., from the intersectional grouping of *White+Rich*) that are, for example, coded as positive (vs. negative).

# **Study 2: Robustness across variations in group lists**

Demonstrating further robustness, the primary findings on frequency of trait stereotypes remain consistent regardless of whether we represent the social groups as shortened lists of 4 words only (shown below) or as the full lists of 12 words (shown in the main text). By “generally” robust we mean that the same dominance of *White, Rich, Male* appears for the primary model (GloVe 840B) and across most other models, although there are some idiosyncrasies.

For instance, using the 4-word lists, the results from *fastText Wiki 2M* show relatively higher frequencies of traits for *Female+Poor* than for *Male+Rich*, and for *Female+White* than for *Male+White*. Such results may suggest that, for some modelling specifications, static embedding models return results more in line with “markedness” of non-default groups. As discussed in the main text, and as seen most often in the contextualized setting of language, “markedness” means that most non-default groups (e.g., *poor women*, *Black women*) require more description because of their non default status and thus appear to be associated with more traits.

Nevertheless, as seen in Figure S5, for the primary model of *GloVe 840B*, even the specific traits classifications are relatively robust across variations representing groups with 4 or 12 words. That is, in Figure S5, most of the traits in the top right quadrant are, as expected, dark blue (indicating that they were classified by both approaches as White+Rich), and so on for all other quadrants as well. Moreover, there is clear consistency when to comes to the qualities of the traits associated with each quadrant: traits associated with the White+Rich quadrant are, as before, strongly positive (94% are positive), while the traits associated with Black+Poor are strongly negative (89% are negative).

***Table S15.***

Percentage of traits, with list of 100 traits, but **shortened group lists of 4 words only,** for each intersectional grouping (quadrant) by embedding method (non z-scored)

|  | ***Static embeddings*** | | | ***Contextualized embeddings*** | | |
| --- | --- | --- | --- | --- | --- | --- |
| ***Intersectional grouping*** | ***GloVe CC 840B*** | ***GloVe Wiki 6B*** | ***fastText Wiki 2M*** | ***Single words with template*** | ***Pooled words with template*** | ***Pooled words no template*** |
| **White + Rich** | **37** | 28 | 32 | **54** | **67** | **52** |
| **White + Poor** | 20 | **36** | 24 | 35 | 28 | 29 |
| **Black + Rich** | 12 | 16 | 9 | 2 | 0 | 6 |
| **Black + Poor** | 31 | 20 | **35** | 9 | 5 | 13 |
| **Male + Rich** | **34** | 34 | 15 | **46** | **52** | 25 |
| **Male + Poor** | 26 | 18 | 23 | 16 | 11 | 9 |
| **Female + Rich** | 15 | 10 | 26 | 10 | 15 | **33** |
| **Female + Poor** | 25 | **38** | **36** | 28 | 22 | 33 |
| **Male + White** | **35** | 31 | 23 | **58** | **62** | 31 |
| **Male + Black** | 25 | 21 | 15 | 4 | 1 | 3 |
| **Female + White** | 22 | **33** | **33** | 31 | 33 | **50** |
| **Female + Black** | 18 | 15 | 29 | 7 | 4 | 16 |

***Note.*** See note to Table S8.

***Table S16.***

Relative percentages of types of traits (by valence, warmth and competence) within each intersectional quadrant for **GloVe 840B embeddings, with shortened group lists**

|  | **Valence** | | **Warmth** | | **Competence** | | **Word Arousal** | | **Word Dominance** | |
| --- | --- | --- | --- | --- | --- | --- | --- | --- | --- | --- |
| **Intersectional grouping** | **Positive** | **Negative** | **Warm** | **Cold** | **Comp** | **Incomp** | **High** | **Low** | **High** | **Low** |
| **White + Rich** | 0.94 | 0.06 | 0.97 | 0.03 | 0.94 | 0.06 | 0.31 | 0.69 | 0.91 | 0.09 |
| **White + Poor** | 0.21 | 0.79 | 0.21 | 0.79 | 0.29 | 0.71 | 0.64 | 0.36 | 0.21 | 0.79 |
| **Black + Rich** | 0.72 | 0.28 | 0.72 | 0.28 | 0.72 | 0.28 | 0.61 | 0.39 | 0.72 | 0.28 |
| **Black + Poor** | 0.11 | 0.89 | 0.11 | 0.89 | 0.11 | 0.89 | 0.56 | 0.44 | 0.11 | 0.89 |
| **Male + Rich** | 0.87 | 0.13 | 0.87 | 0.13 | 0.87 | 0.13 | 0.29 | 0.71 | 0.87 | 0.13 |
| **Male + Poor** | 0.13 | 0.87 | 0.13 | 0.87 | 0.13 | 0.87 | 0.65 | 0.35 | 0.13 | 0.87 |
| **Female + Rich** | 0.84 | 0.16 | 0.89 | 0.11 | 0.84 | 0.16 | 0.63 | 0.37 | 0.79 | 0.21 |
| **Female + Poor** | 0.15 | 0.85 | 0.15 | 0.85 | 0.19 | 0.81 | 0.52 | 0.48 | 0.15 | 0.85 |
| **Male + White** | 0.85 | 0.15 | 0.85 | 0.15 | 0.85 | 0.15 | 0.30 | 0.70 | 0.85 | 0.15 |
| **Male + Black** | 0.26 | 0.74 | 0.26 | 0.74 | 0.26 | 0.74 | 0.59 | 0.41 | 0.26 | 0.74 |
| **Female + White** | 0.53 | 0.47 | 0.58 | 0.42 | 0.58 | 0.42 | 0.58 | 0.42 | 0.47 | 0.53 |
| **Female + Black** | 0.37 | 0.63 | 0.37 | 0.63 | 0.37 | 0.63 | 0.56 | 0.44 | 0.37 | 0.63 |

*Note.* Each number reflects the percentage of traits from the given quadrant (e.g., from the intersectional grouping of *White+Rich*) that are, for example, coded as positive (vs. negative).

| **A.** | ** |
| --- | --- |
| **B.** | ** |
| **C.** | ** |
|  |  |

**Fig S5. Scatterplots of classifications across variations in group lists.** Colors represent the classifications from the main model (GloVe 840B with lists of 12 words for each group); traits are placed according to classifications from the robustness analysis (GloVe 840B with lists of 4 words for each group). If results of specific trait classifications were identical between modelling variations of group lists, the colors of each quadrant would be consistent (e.g., all traits in the top right quadrant would be colored dark blue). Panel A represents Gender-by-race intersectional quadrants; Panel B is Gender-by-class; Panel C is Race-by-class.

# **Study 2: Robustness across nouns and verbs**

As a final test of robustness, we also investigate how the frequencies vary when we use a list of nouns or verbs (rather than trait adjectives). As with the variations in results for the different group lists (4 versus 12 words), results for the top 100 most positive/negative nouns show generally similar results as with the primary model (GloVe 840B with 100 traits). As before, *White, Rich, and Male* show general dominance of overall frequencies, albeit with some idiosyncracies for the other static models. For instance, once again the *fastText Wiki 2M* setting indicates relatively higher frequencies of nouns and verbs associated with the *Black+Poor, Female+Poor*, and *Female+Black* quadrants, all categories that are the most “non-default” and therefore perhaps the most marked in language.

Nevertheless, as before, the features of the 100 nouns associated with each quadrant shows strong consistency with the 100 trait analyses: for example, 94% of the nouns associated with the White+Rich quadrant are positive; 87% of the nouns associated with the Black+Poor quadrant are negative. Similarly, the features of the 100 verbs associated with each quadrant show consistent patterns of valence imbalances: 74% of verbs associated with White+Rich quadrant are positive; 83% of verbs associated with Black+Poor quadrant are negative.

***Table S17.***

Percentage of traits, with list of **100 nouns** for each intersectional grouping (quadrant) by embedding method (non z-scored)

|  | ***Static embeddings*** | | | ***Contextualized embeddings*** | | |
| --- | --- | --- | --- | --- | --- | --- |
| ***Intersectional grouping*** | ***GloVe CC 840B*** | ***GloVe Wiki 6B*** | ***fastText Wiki 2M*** | ***Single words with template*** | ***Pooled words with template*** | ***Pooled words no template*** |
| **White + Rich** | **46** | 26 | 19 | **63** | **77** | 2 |
| **White + Poor** | 35 | **41** | 24 | 20 | 11 | **85** |
| **Black + Rich** | 17 | 5 | 10 | 0 | 1 | 1 |
| **Black + Poor** | 2 | 27 | **47** | 17 | 11 | 12 |
| **Male + Rich** | 23 | 16 | 12 | 13 | 32 | 2 |
| **Male + Poor** | 25 | 20 | 26 | 16 | 5 | 24 |
| **Female + Rich** | 25 | 15 | 17 | **50** | **46** | 1 |
| **Female + Poor** | 27 | **48** | **45** | 21 | 17 | **73** |
| **Male + White** | 37 | 27 | 14 | 20 | 33 | 21 |
| **Male + Black** | 11 | 9 | 24 | 9 | 4 | 5 |
| **Female + White** | **44** | **40** | 29 | **63** | **55** | **66** |
| **Female + Black** | 8 | 23 | **33** | 8 | 8 | 8 |

***Note.*** See note to Table S8.

***Table S18.***

Relative percentages of types of traits (by valence, arousal, dominance) within each intersectional quadrant for **GloVe 840B embeddings, with 100 nouns**

|  | **Valence** | | **Word Arousal** | | **Word Dominance** | |
| --- | --- | --- | --- | --- | --- | --- |
| **Intersectional grouping** | **Positive** | **Negative** | **High** | **Low** | **High** | **Low** |
| **White + Rich** | 0.94 | 0.06 | 0.47 | 0.53 | 0.94 | 0.06 |
| **White + Poor** | 0.37 | 0.63 | 0.47 | 0.53 | 0.37 | 0.63 |
| **Black + Rich** | 0.41 | 0.59 | 0.88 | 0.12 | 0.41 | 0.59 |
| **Black + Poor** | 0.13 | 0.87 | 0.33 | 0.67 | 0.13 | 0.87 |
| **Male + Rich** | 0.64 | 0.36 | 0.68 | 0.32 | 0.64 | 0.36 |
| **Male + Poor** | 0.29 | 0.71 | 0.33 | 0.67 | 0.29 | 0.71 |
| **Female + Rich** | 0.88 | 0.12 | 0.54 | 0.46 | 0.88 | 0.12 |
| **Female + Poor** | 0.16 | 0.84 | 0.44 | 0.56 | 0.16 | 0.84 |
| **Male + White** | 0.68 | 0.32 | 0.48 | 0.52 | 0.68 | 0.32 |
| **Male + Black** | 0.25 | 0.75 | 0.54 | 0.46 | 0.25 | 0.75 |
| **Female + White** | 0.79 | 0.21 | 0.46 | 0.54 | 0.79 | 0.21 |
| **Female + Black** | 0.22 | 0.78 | 0.52 | 0.48 | 0.22 | 0.78 |

*Note.* Each number reflects the percentage of nouns from the given quadrant (e.g., from the intersectional grouping of *White+Rich*) that are, for example, coded as positive (vs. negative). Note that the nouns were not projected onto the competence and warmth axes and therefore do not have the relative frequencies for those dimensions.

***Table S19.***

Percentage of traits, with list of **100 verbs** for each intersectional grouping (quadrant) by embedding method (non z-scored)

|  | ***Static embeddings*** | | | ***Contextualized embeddings*** | | |
| --- | --- | --- | --- | --- | --- | --- |
| ***Intersectional grouping*** | ***GloVe CC 840B*** | ***GloVe Wiki 6B*** | ***fastText Wiki 2M*** | ***Single words with template*** | ***Pooled words with template*** | ***Pooled words no template*** |
| **White + Rich** | **42** | 30 | 24 | **58** | **73** | 37 |
| **White + Poor** | 34 | 32 | 15 | 25 | 14 | **41** |
| **Black + Rich** | 7 | 2 | 17 | 0 | 0 | 4 |
| **Black + Poor** | 17 | **36** | **44** | 17 | 13 | 18 |
| **Male + Rich** | **29** | 20 | 20 | 25 | 31 | 14 |
| **Male + Poor** | 24 | 20 | 21 | 17 | 7 | 18 |
| **Female + Rich** | 20 | 12 | 21 | **33** | **42** | 27 |
| **Female + Poor** | 27 | **48** | **38** | 25 | 20 | **41** |
| **Male + White** | **40** | **36** | 21 | 33 | 36 | 25 |
| **Male + Black** | 13 | 4 | 20 | 9 | 2 | 7 |
| **Female + White** | 36 | 26 | 18 | **50** | **51** | **53** |
| **Female + Black** | 11 | 34 | **41** | 8 | 11 | 15 |

***Note.*** See note to Table S8.

***Table S20.***

Relative percentages of types of traits (by valence, arousal, dominance) within each intersectional quadrant for **GloVe 840B embeddings, with 100 verbs**

|  | **Valence** | | **Word Arousal** | | **Word Dominance** | |
| --- | --- | --- | --- | --- | --- | --- |
| **Intersectional grouping** | **Positive** | **Negative** | **High** | **Low** | **High** | **Low** |
| **White + Rich** | 0.74 | 0.26 | 0.52 | 0.48 | 0.77 | 0.23 |
| **White + Poor** | 0.48 | 0.52 | 0.43 | 0.57 | 0.48 | 0.52 |
| **Black + Rich** | 0.69 | 0.31 | 0.56 | 0.44 | 0.62 | 0.38 |
| **Black + Poor** | 0.17 | 0.83 | 0.50 | 0.50 | 0.17 | 0.83 |
| **Male + Rich** | 0.59 | 0.41 | 0.48 | 0.52 | 0.62 | 0.38 |
| **Male + Poor** | 0.22 | 0.78 | 0.57 | 0.43 | 0.22 | 0.78 |
| **Female + Rich** | 0.94 | 0.06 | 0.61 | 0.39 | 0.89 | 0.11 |
| **Female + Poor** | 0.37 | 0.63 | 0.40 | 0.60 | 0.37 | 0.63 |
| **Male + White** | 0.53 | 0.47 | 0.50 | 0.50 | 0.56 | 0.44 |
| **Male + Black** | 0.25 | 0.75 | 0.55 | 0.45 | 0.25 | 0.75 |
| **Female + White** | 0.77 | 0.23 | 0.45 | 0.55 | 0.77 | 0.23 |
| **Female + Black** | 0.42 | 0.58 | 0.50 | 0.50 | 0.38 | 0.62 |

*Note.* Each number reflects the percentage of traits from the given quadrant (e.g., from the intersectional grouping of *White+Rich*) that are, for example, coded as positive (vs. negative). Note that the verbs were not projected onto the competence and warmth axes and therefore do not have the relative frequencies for those dimensions.

| **A.**  **** | **D. ** |
| --- | --- |
| **B.** | **E.** |
| **C.** | **F.** |
|  |  |

**Fig S6. Scatterplots of nouns and verbs across intersectional quadrants for GloVe CC 840B.** Panels A and D represents Gender-by-race intersectional quadrants, for nouns and verbs, respectively; Panel B and E represent Gender-by-class for nouns and verbs, respectively; Panel C and F represent Race-by-class for nouns and verbs, respectively.
